# Supplementary material for: Extracellular matrix educates an immunoregulatory tumor macrophage phenotype found in ovarian cancer metastasis
Source: Nat Commun. 2023 May 15;14:2514. doi: 10.1038/s41467-023-38093-5 (PMC10185550; doi:10.1038/s41467-023-38093-5)
Supplement: Supplementary file 1 — Supplementary Information [file 41467_2023_38093_MOESM1_ESM.pdf]

Extracellular matrix educates an immunoregulatory tumor macrophage phenotype found in ovarian cancer metastasis

E. H. Puttock[1#], E. J. Tyler[1#], M. Manni[2], E. Maniati[1], C. Butterworth,[1] M. Burger Ramos[1], E. Peerani[1], P. Hirani[1], V. Gauthier[1], Y. Liu[1], G. Maniscalco[1], V. Rajeeve[1], P. Cutillas[1], C. Trevisan[3], M. Pozzobon[3], M. Lockley[1], J. Rastrick[4], H. Läubli[2], A. White[4], & O. M. T. Pearce[1*]

[1]Queen Mary University of London, Barts Cancer Institute, John Vane Science Centre, London, UK, EC1M 6BQ

[2]Department of Biomedicine and Division of Medical Oncology, University Hospital Basel, Hebelstrasse 20, 4031 Basel, Switzerland

[3] Department of Women and Children Health, University of Padova and Fondazione Istituto di Ricerca Pediatrica Città della Speranza, Corso Stati Uniti 4, 35127 Padova

[4]UCB Pharma Ltd, 208 Bath Road, Slough, Berkshire, SL1 3WE

#These authors contributed equally

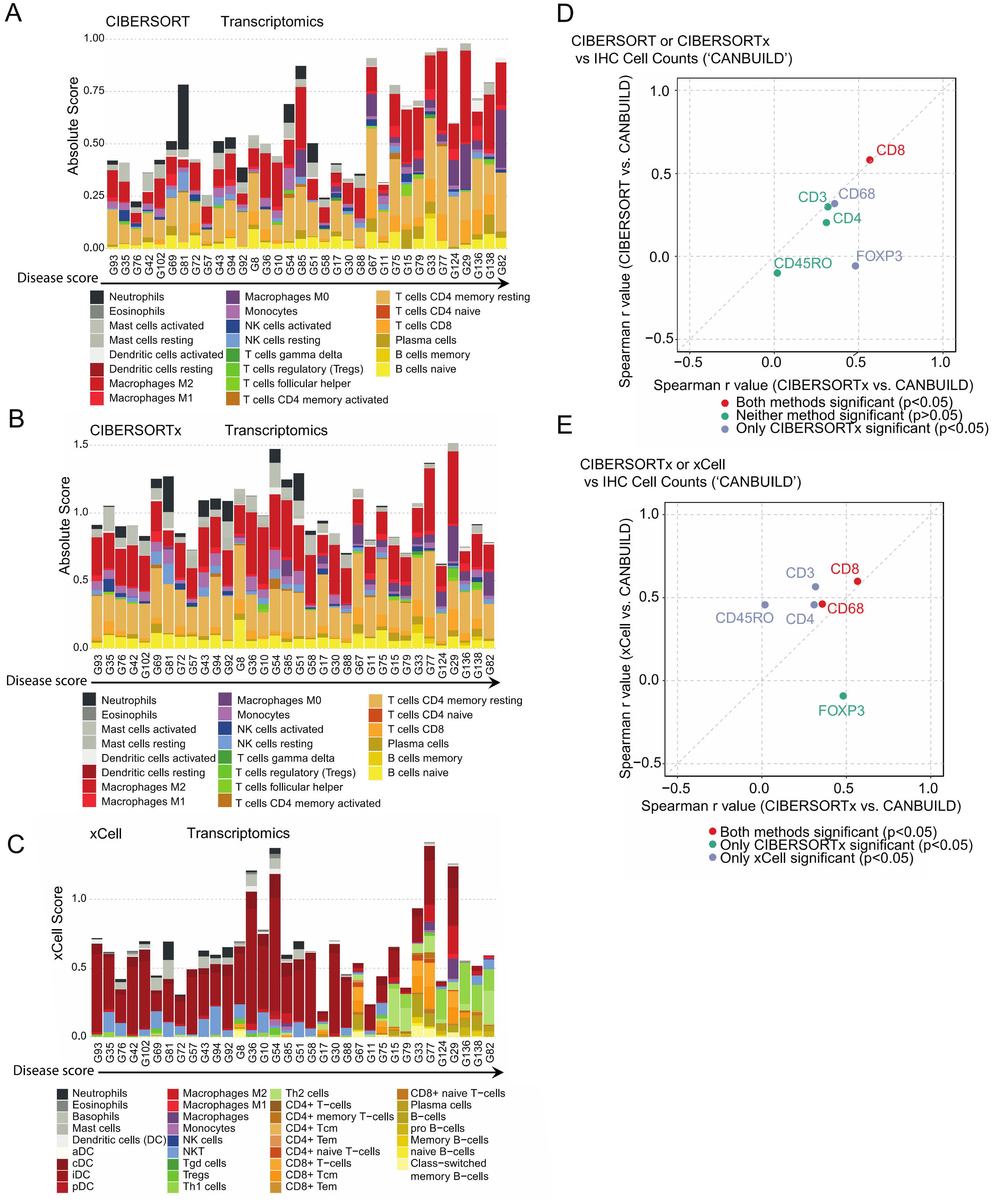

Supplementary Figure 1. Validation of computational algorithms predicting transcriptomic immune landscape in HGSOC. A-C) Stacked bar charts of immune cell predictions by CIBERSORT, CIBERSORTx, and xCell respectively, ordered by sample disease score. Cell color codes are presented in a key below with color matching across methods. **D-E)** Scatter plots of **D)** CIBERSORTx and CIBERSORT or **E)** CIBERSORTx and xCell correlations with previous immunohistochemistry staining cell counts using six immune markers present in CANBUILD dataset, in human HGSOC tissues. Regression analysis of summed abundances of all possible predicted immune cell types with each marker was completed to generate the plotted r values. Points are colored based on the significance of their r values from each technique, where p < 0.05 is significant. Spearman's regression analysis with two-sided alternative hypothesis testing was completed to generate plotted r values, N = 32 HGSOC samples.

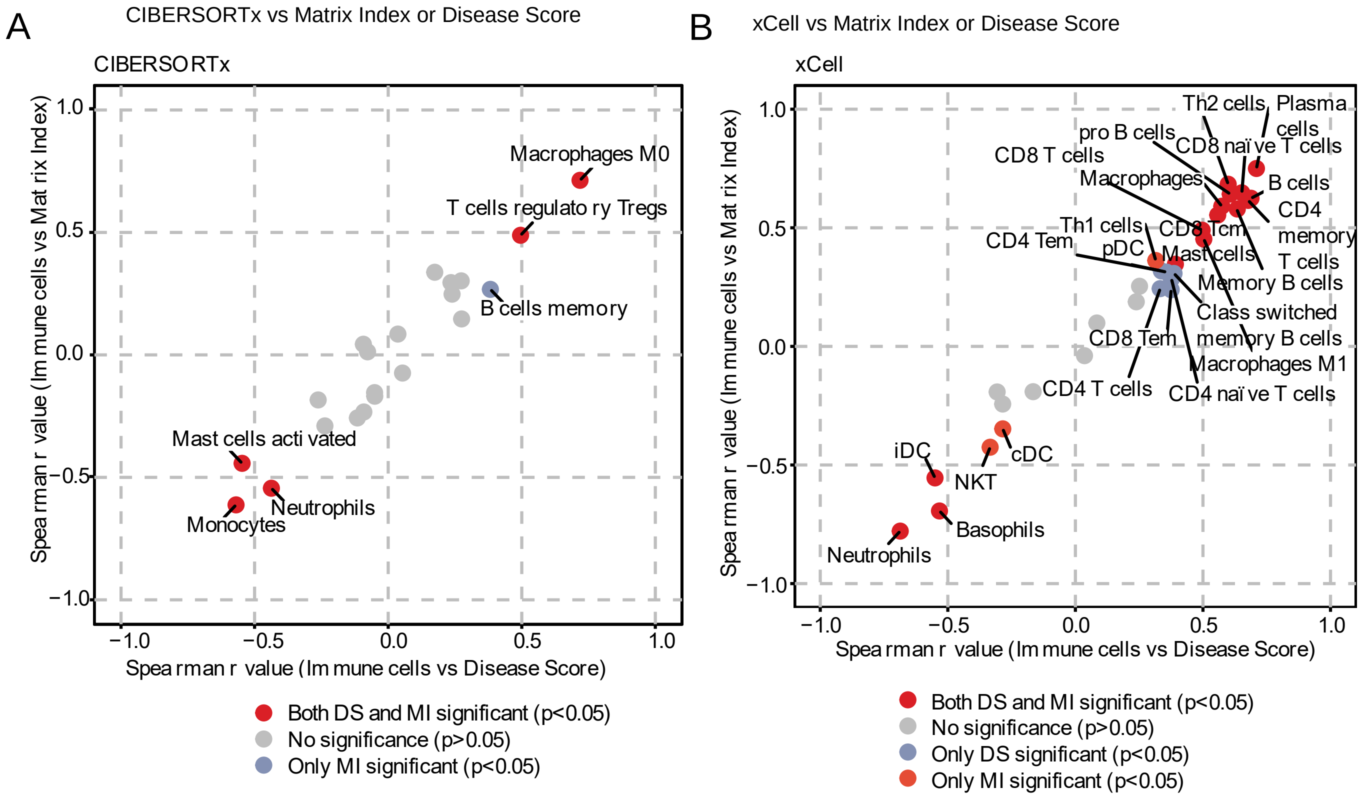

Supplementary Figure 2. M0 macrophages significantly correlate with MI and disease score. A) Scatter plot of immune cell estimates using CIBERSORTx, correlated to sample disease scores (DS) and matrix index (MI). Spearman's regression analysis was completed to generate the plotted r values. Points are colored based on the significance of their r values as depicted in the key, where $p<0.05$ is significant. Spearman's regression analysis was completed to generate plotted r values, N = 32 HGSOC samples. **B)** Scatter plots of immune cell estimates using xCell, correlated to sample disease scores (DS) and matrix index (MI). Spearman's regression analysis was completed to generate the plotted r values. Points are colored based on the significance of their r values as depicted in the key, where $p<0.05$ is significant. Spearman's regression analysis with two-sided alternative hypothesis testing was completed to generate plotted r values, N = 32 HGSOC samples.

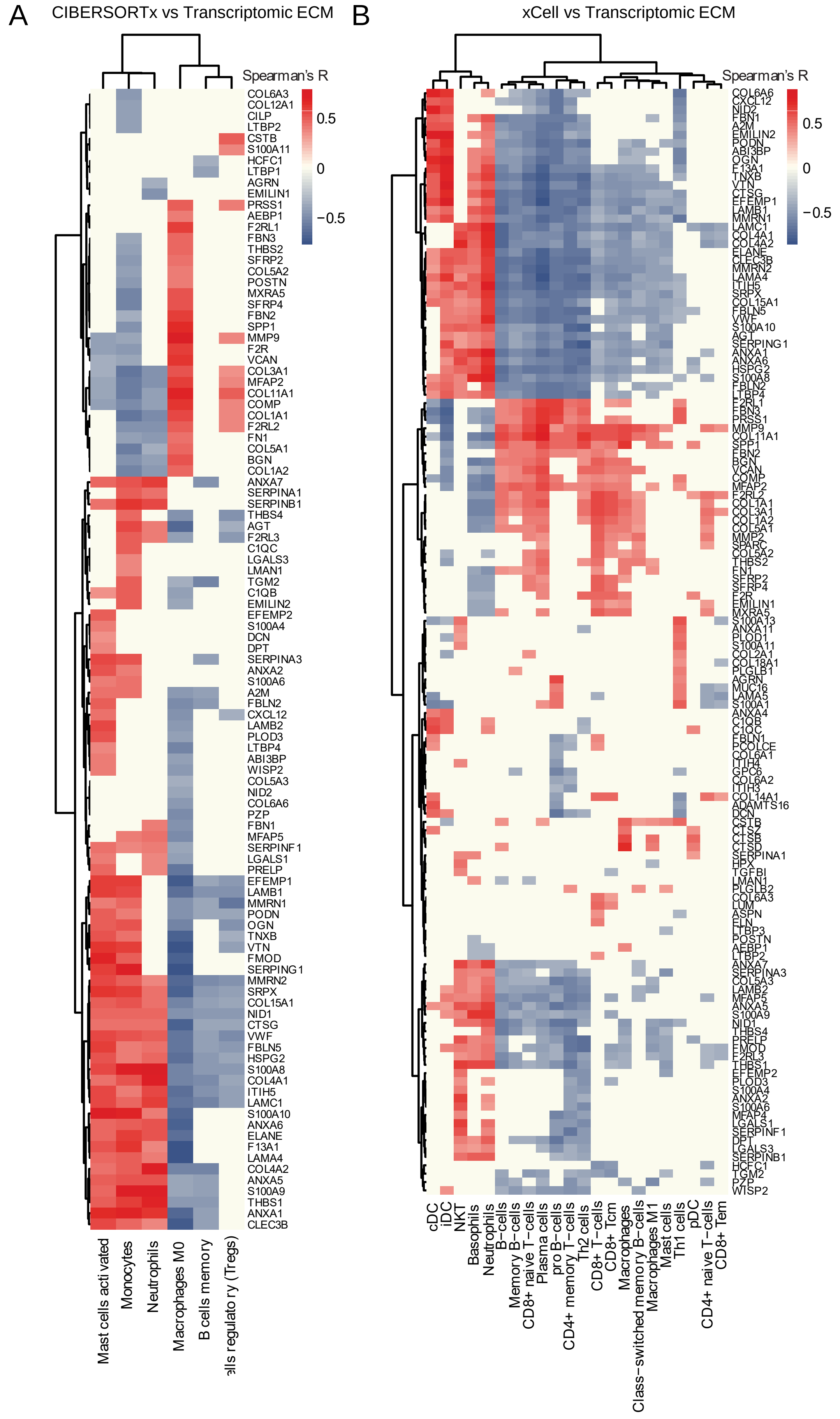

Supplementary Figure 3. M0 macrophages significantly correlate with a matrisome signature at the transcriptomic level for both CIBERSORTx and xCell. A-B) Heatmap of ECM genes significantly associated with significant immune cells from **A)** CIBERSORTx and **B)** xCell analysis. Spearman's r values with two-sided alternative hypothesis testing representing correlation between immune abundances and gene expression levels are plotted according to the color scale, with positive correlations in red and negative in blue. Cream colored associations are insignificant with a correlation $p > 0.05$, N = 32 HGSOC samples. Ordered by unsupervised clustering.

CIBERSORTx or xCell vs Transcriptomic and Proteomic ECM

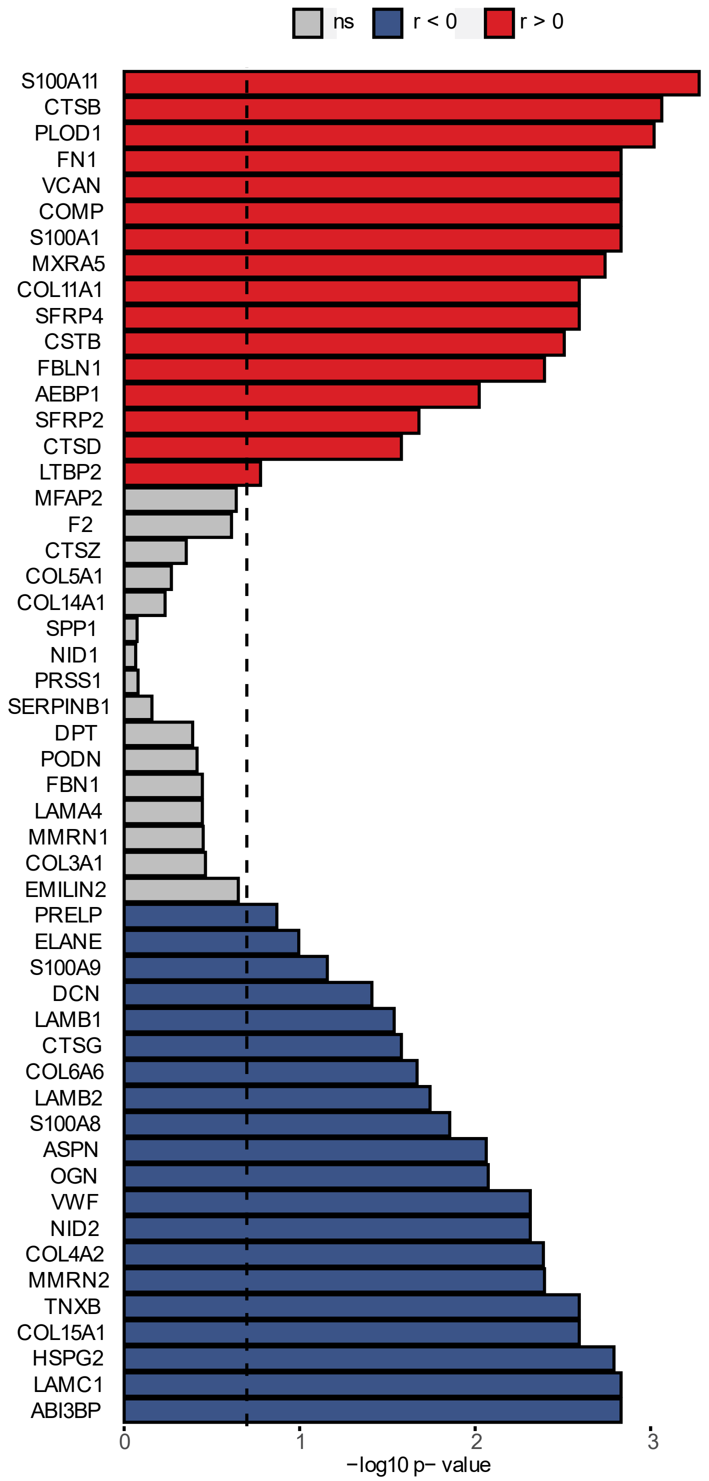

Supplementary Figure 4. Identification of significant ECM proteins associated to disease score. Barplot illustrates Pearson *p*-values, FDR corrected using the Benjamini & Hochberg method. The dotted line specifies the significance cut-off p = 0.2. All proteins listed were previously identified to have significant associations with immune subsets, at both the gene and protein level. * indicates proteins forming part of the HGSOC matrix index. p values correspond to Pearson's method with two-sided alternative hypothesis testing, N = 32 HGSOC samples.

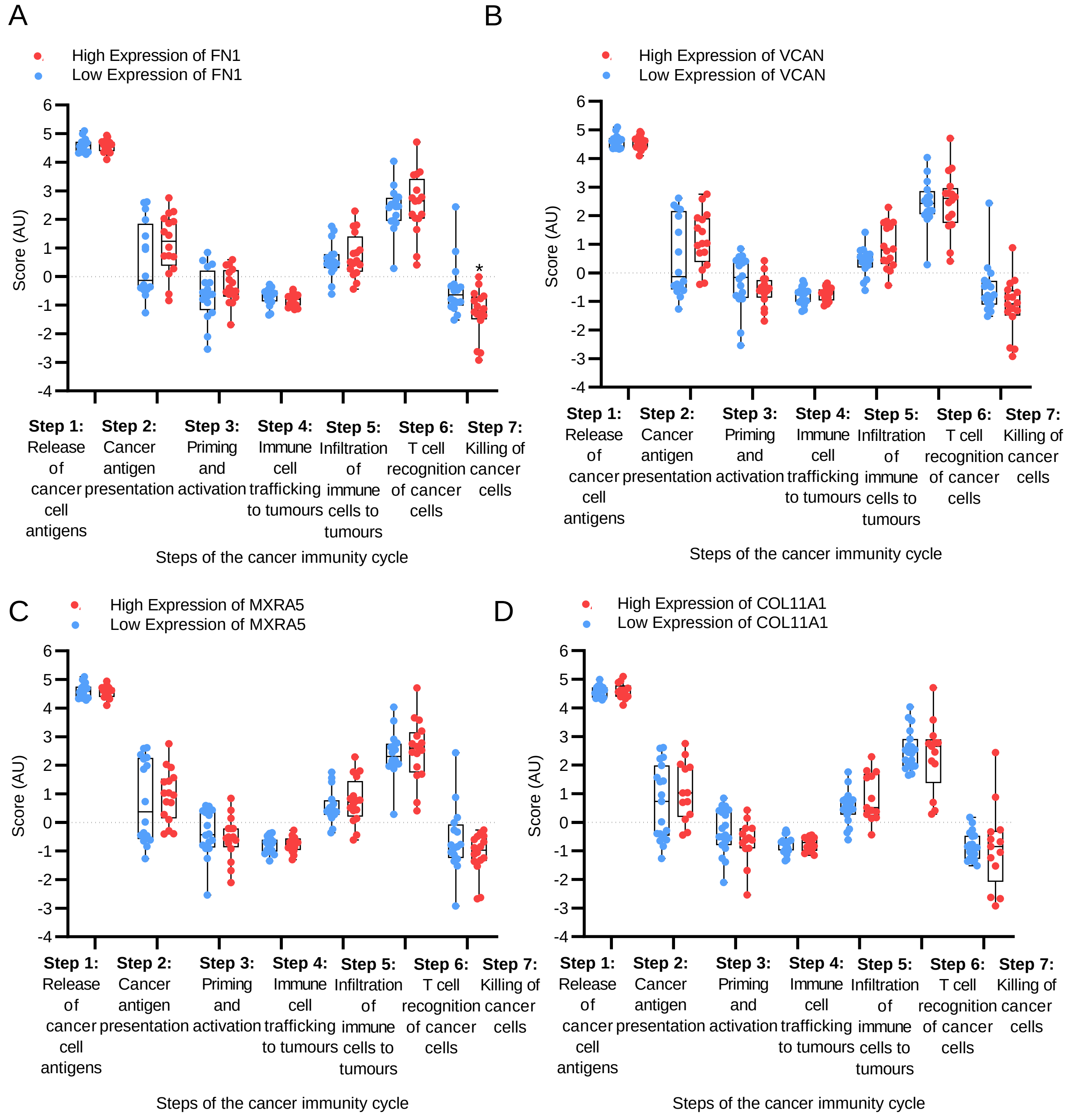

Supplementary Figure 5. Boxplot of predicted tumor immune phenotype stage scores by TIP. HGSOC samples separated by high (red) and low (blue) protein expression levels of **A)** FN1, **B)** VCAN, **C)** COL11A1, **D)** MXRA5, **E)** SFRP2. Scores are based upon expression of signature genes and represent activity levels. N = 32. Within each box, the centre line denotes the median value (50th percentile) while the box extends from the 25th to the 75th percentile; whiskers mark the maximum and minimum values. Two-way ANOVA significance between each group is presented as *p = 0.0213. N = 32 HGSOC samples (16 low and 16 high).

E

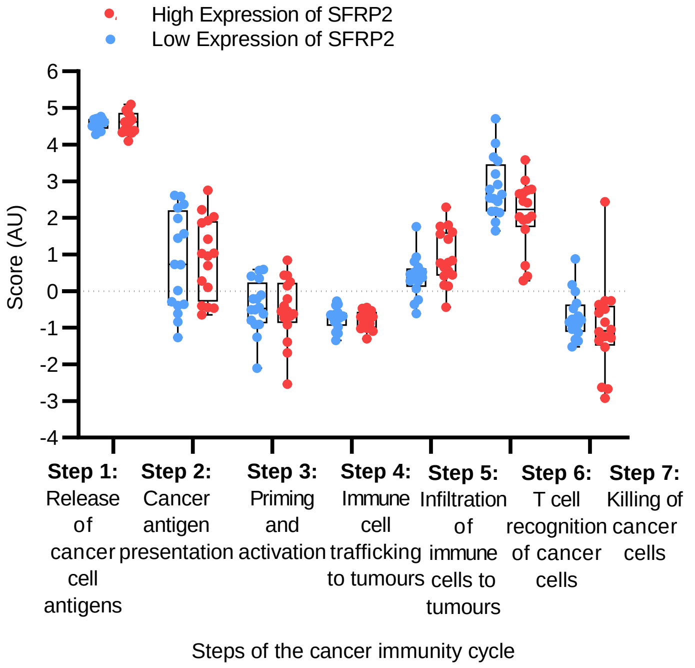

Supplementary Figure 5. Boxplot of predicted tumor immune phenotype stage scores by TIP. HGSOC samples separated by high (red) and low (blue) protein expression levels of **A)** FN1, **B)** VCAN, **C)** COL11A1, **D)** MXRA5, **E)** SFRP2. Scores are based upon expression of signature genes and represent activity levels. N = 32. Within each box, the centre line denotes the median value (50th percentile) while the box extends from the 25th to the 75th percentile; whiskers mark the maximum and minimum values. Two-way ANOVA significance between each group is presented as *p = 0.0213. N = 32 HGSOC samples (16 low and 16 high).

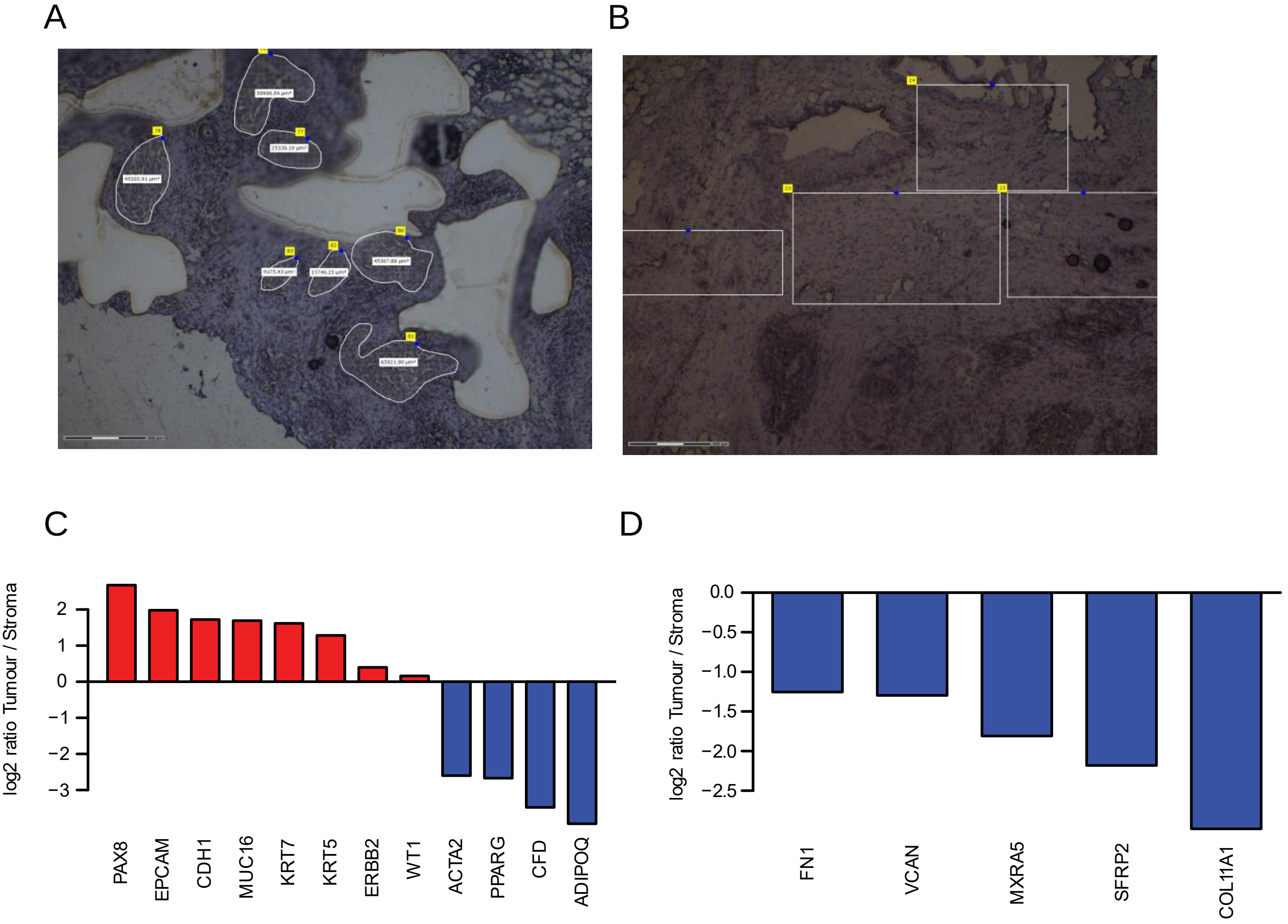

Supplementary Figure 6. Laser capture demonstrates enrichment for matrisome molecules associated with M0 macrophages in the stroma. Representative images of tissue with areas marked for **A)** tumor and **B)** stroma laser capture**. C)** Validation of tumor and stroma area laser capture using known malignant cell and fibroblast markers, e.g. malignant cell markers: PAX8, EPCAM, and fibroblast marker: ACTA2. **D)** Tumor/Stroma ratio for matrisome proteins associated with M0 macrophages. N=2 (G33, G75).

A

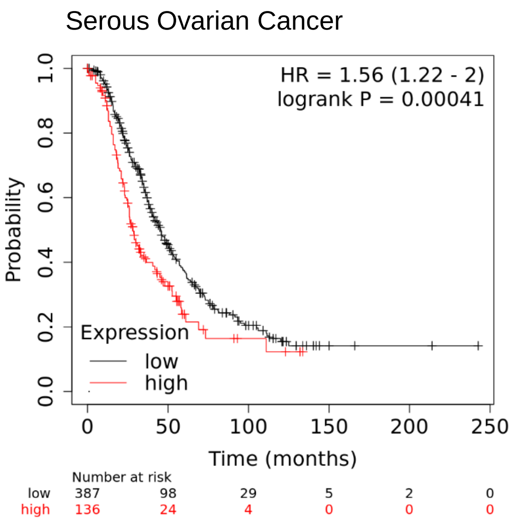

Median Overall Survival

Low expression cohort (months)	High expression cohort (months)
45.63	28.25

B

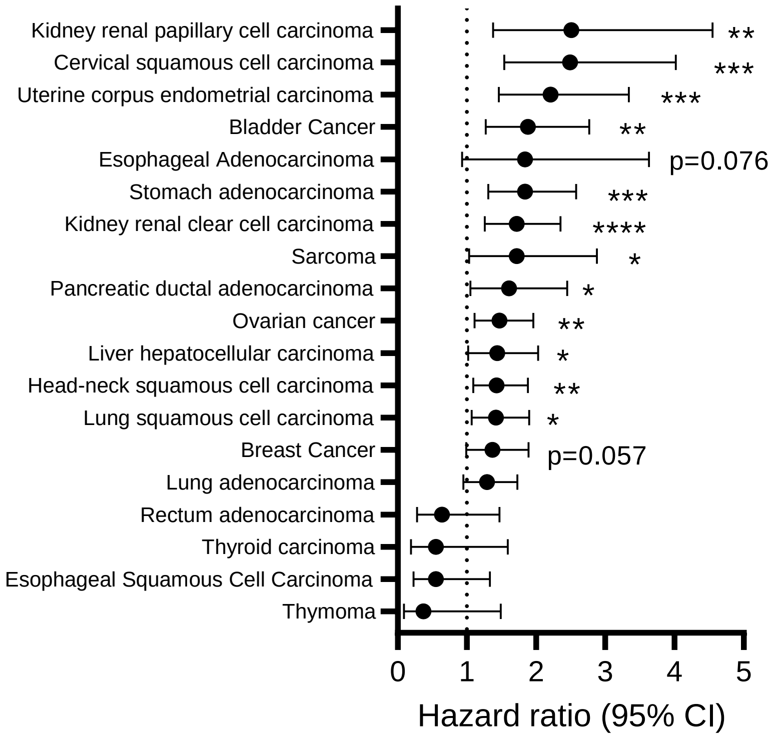

Supplementary Figure 7. M0 macrophage ECM signature associates with poorer prognosis in HGSOC and across multiple cancer types. A) Kaplan-Meier survival curves with overall survival, divided by low and high gene expression levels of the averaged ECM signature associated with M0 macrophages. Serous ovarian cancer patients N = 523. Median survival time is calculated at 50% survival probability. **B)** Multivariate hazard ratio (HR) with 95% CI, derived using multiple datasets across a range of cancer types, with patients divided by low and high gene expression levels of the M0 macrophage-associated ECM signature averaged. HR > 1 indicates that the M0 macrophage-associated ECM signature gene expression is inversely correlated with OS, while HR < 1 shows positively correlated OS. Log ranked p-values significances are presented by asterisks; **** p < 0.0001, ***p < 0.001, **p < 0.01 and *p < 0.05. Data are presented as mean values +/- error bars. KIRP p = 0.0017, N = 288; CESC p = 0.00011, N = 304; UCEC p = 0.00011, N = 543; BLCA p = 0.0012, N = 405; ESCA p = 0.076, N = 80; STAD p = 0.00031, N = 375; KIRC p = 0.0005, N = 530; SARC p = 0.035, N = 259; PDAC p = 0.026, N = 177; OV p = 0.0073, N = 371; LIHC p = 0.039, N = 371; HNSC p = 0.0099, N = 500; LUSC p = 0.016, N = 501; BRCA p = 0.057, N = 1090; LUAD p = 0.099, N = 513; READ p = 0.29, N = 165; THCA p = 0.26, N = 502; ESCC p = 0.18, N = 81; THYM p = 0.15, N = 119.

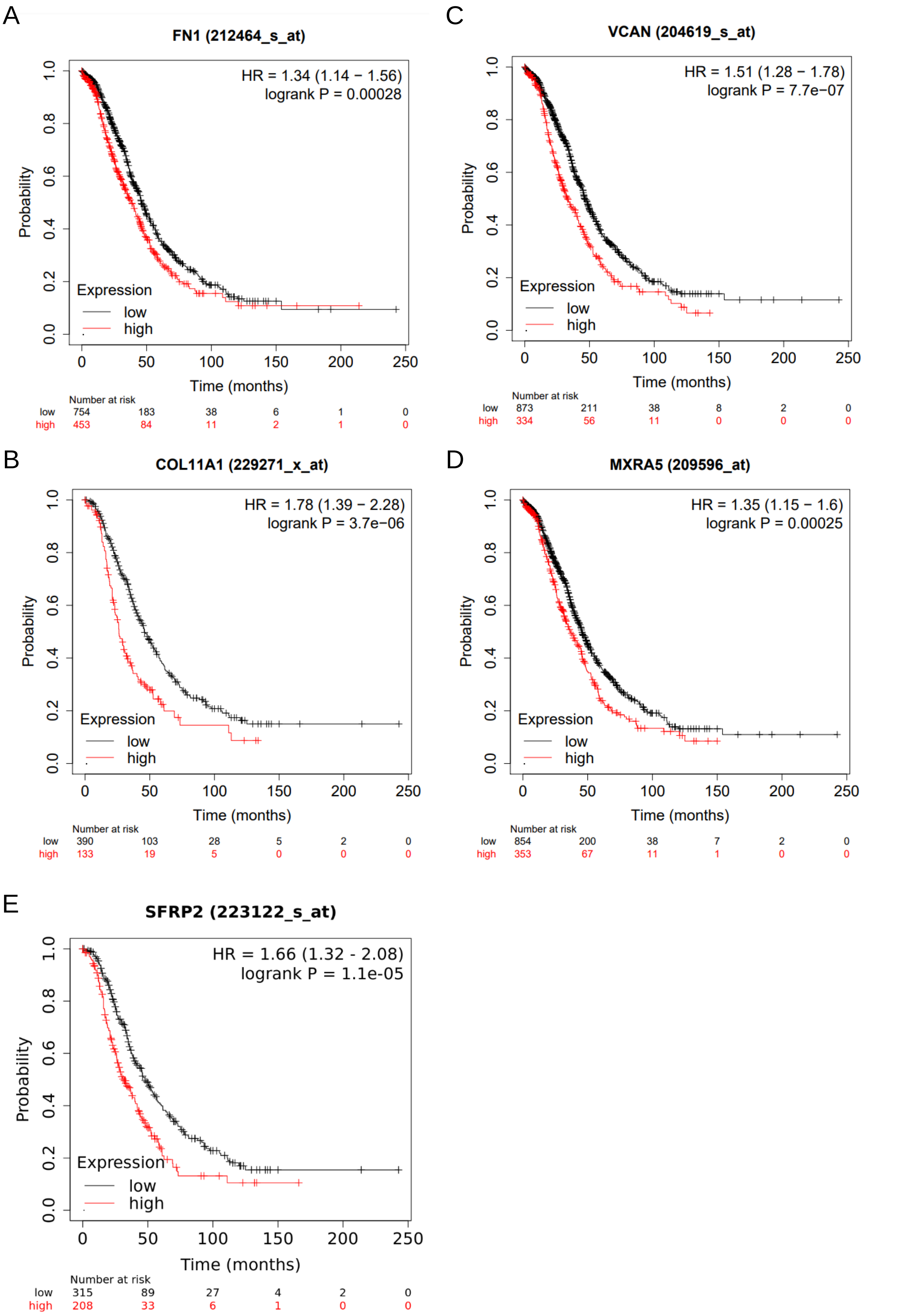

Supplementary Figure 8. Individual Kaplan-Meier survival curves for M0 macrophage associated ECM signature genes. A-E) Kaplan-Meier survival curves for **A)** FN1 **B)** COL11A1 **C)** VCAN **D)** MXRA5, **E)** SFRP2, divided by low and high gene expression levels of each individual gene. Serous ovarian cancer patients N = 523. Median survival time is calculated at 50% survival probability.

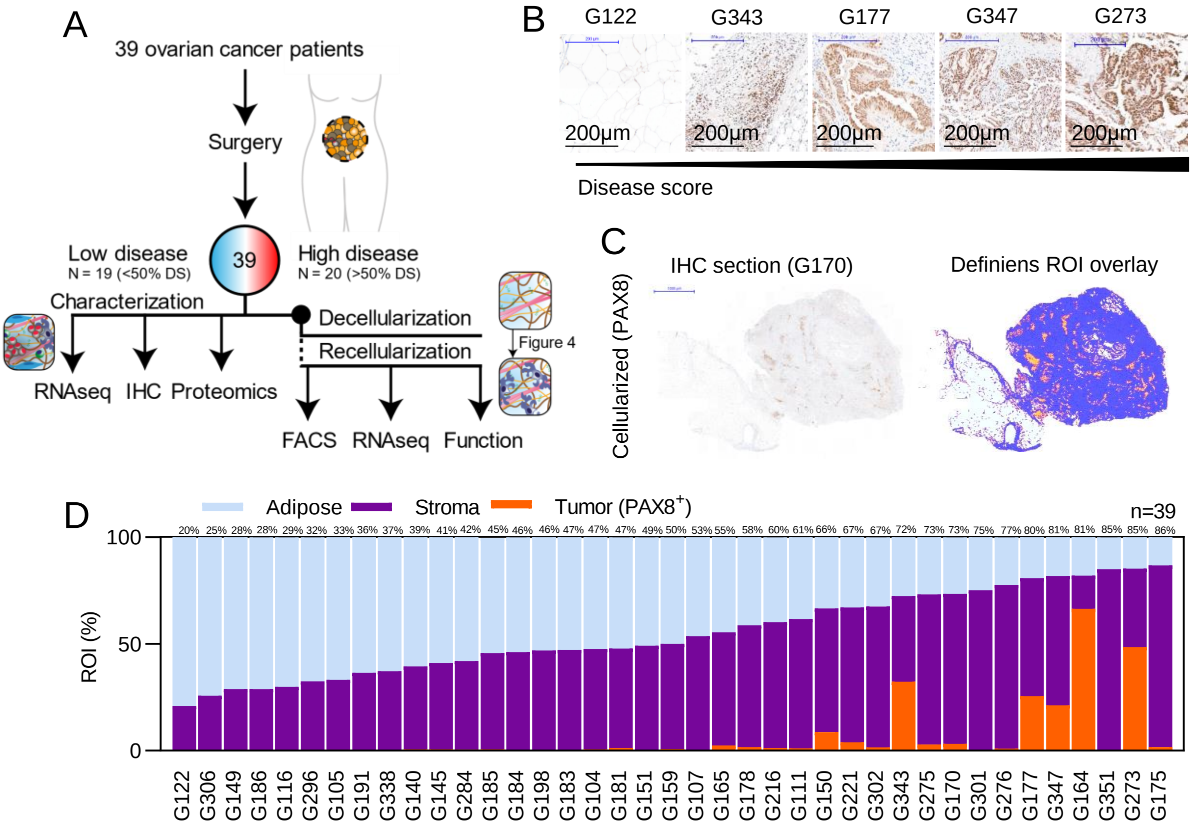

Supplementary Figure 9. Overview of the studied ovarian cancer omental samples and their immune landscape. A) Schematic of ovarian metastatic sample acquisition and analyses. **B)** Representative IHC sections of PAX8 stain increasing with disease score. Scale bar = 200μm. N = 39. **C)** Representative digital analysis using Definiens imaging software (Tissue ID: G170). Scale bar = 1000 μm. **D)** Ovarian cancer metastatic omentum region of interest (ROI) percentage ordered by increasing disease score N = 39.

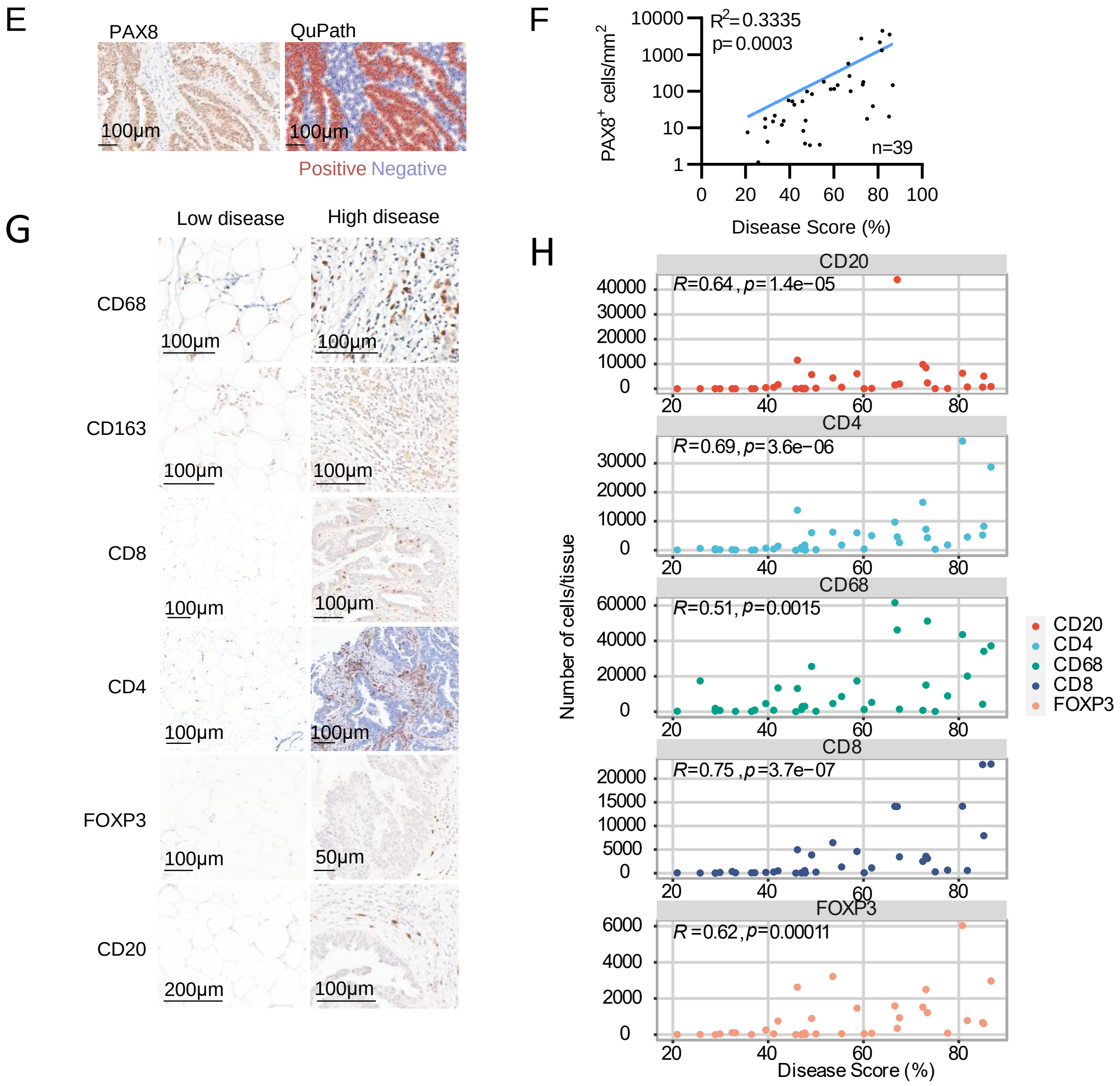

Supplementary Figure 9. Overview of the studied ovarian cancer omental samples and their immune landscape. E) Representative PAX8 stains and matched QuPath digital overlay (Tissue ID: G177). Scale bar = 50µm. N = 39. **F)** Scatter plot of PAX8 cell count by QuPath analysis versus disease score. Least squares regression line. R^2 = 0.3335, p = 0.0003. N = 39. **G)** Representative IHC stains for immune cells in low and high disease tissues. Scale bar = 50-200µm. N = 39. **H)** Cleveland plots of IHC staining for CD68 (N = 38), CD163 (N = 38), CD20 (N = 38), CD3 (N = 38), CD4 (N = 38), CD8 (N = 38) and FOXP3 (N = 38) (cells/ tissue). Spearman's regression analysis with two-sided alternative hypothesis testing was completed to generate plotted r values.

A

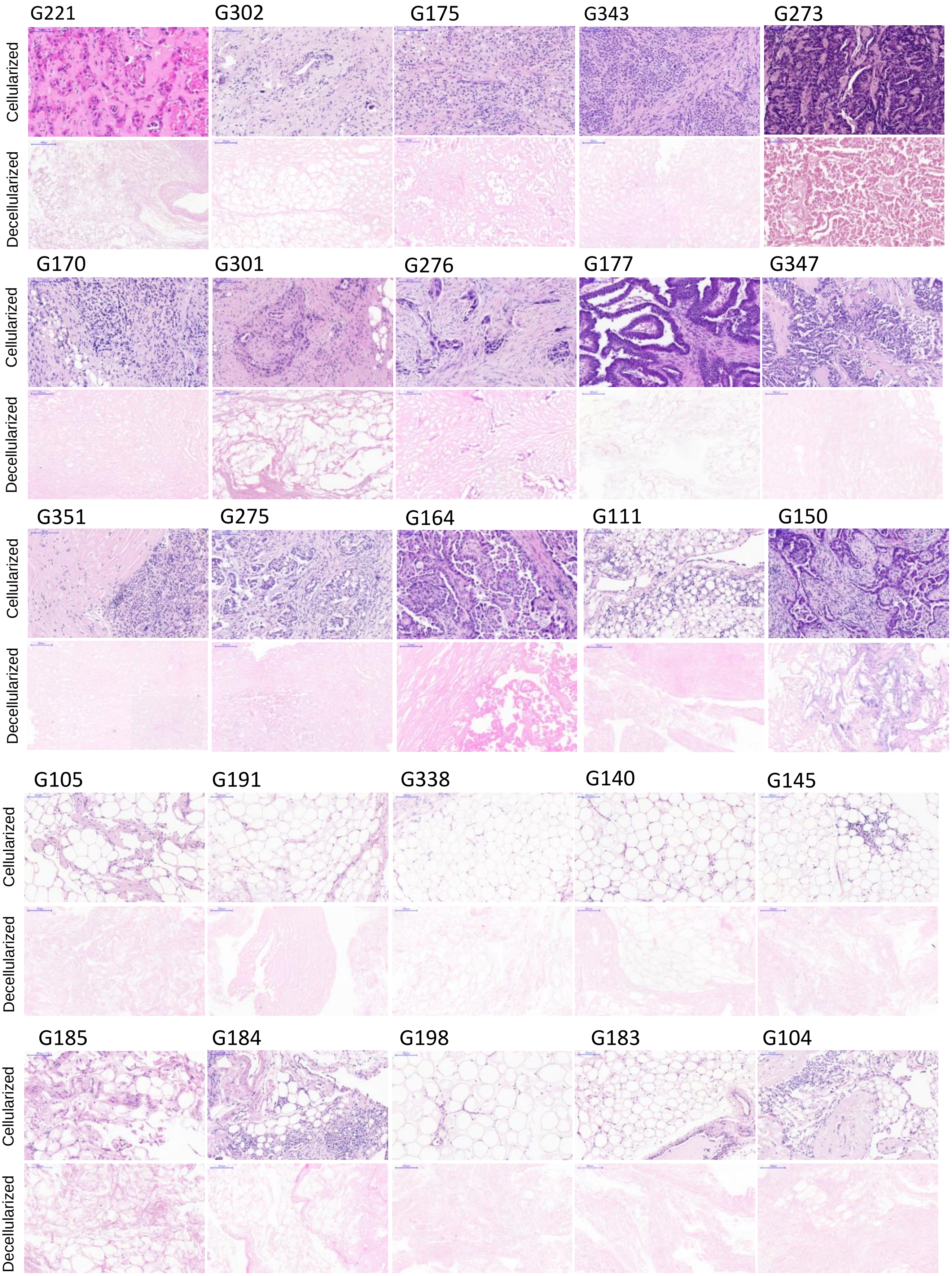

Supplementary Figure 10. H&E stains of cellularized and decellularized tissues from **A-B)** low, medium and high diseased ovarian cancer omental samples. Scale bar = 100 µm. N = 39.

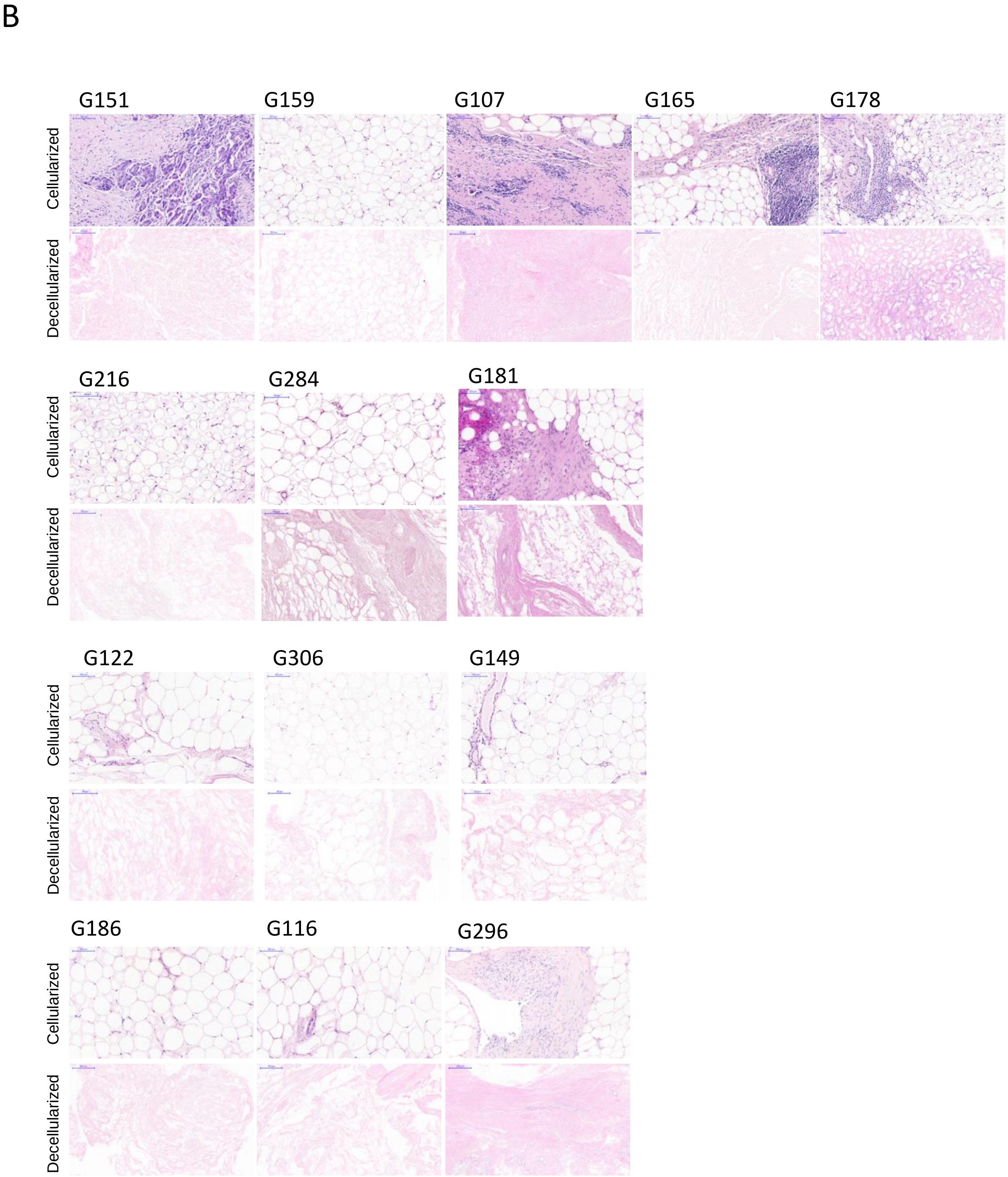

Supplementary Figure 10. H&E stains of cellularized and decellularized tissues from **A-B)** low, medium and high diseased ovarian cancer omental samples. Scale bar = 100 µm. N = 39.

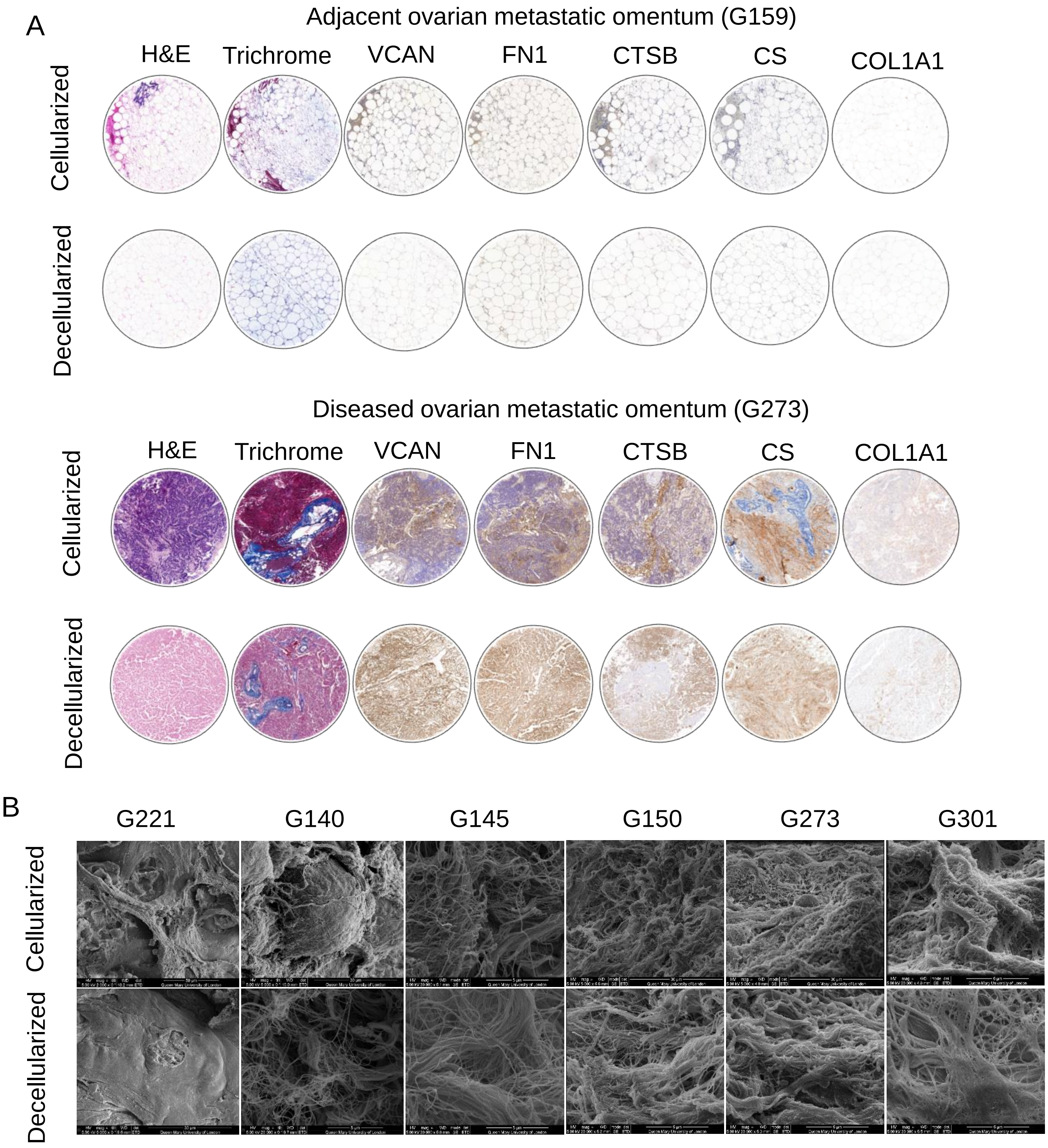

Supplementary Figure 11. Representative sections of IHC stained low (G159) and high diseased (G273) cellularized and decellularized ovarian cancer samples. A) Stains included anti-VCAN (polyclonal, Ab202906; 1:200), anti-COL1A1 (polyclonal, HPA011795; 1:500), anti-FN1 (polyclonal, Ab23750; 1:500), anti-CTSB (ab125067; 1:50), from Abcam, anti-CS (clone CS-56, Ab11570; 1:600). **B)** Scanning Electron Microscopy (SEM) images of matched cellularized and decellularized omentum samples. Scale bars are indicated on each image. N = 6 each.

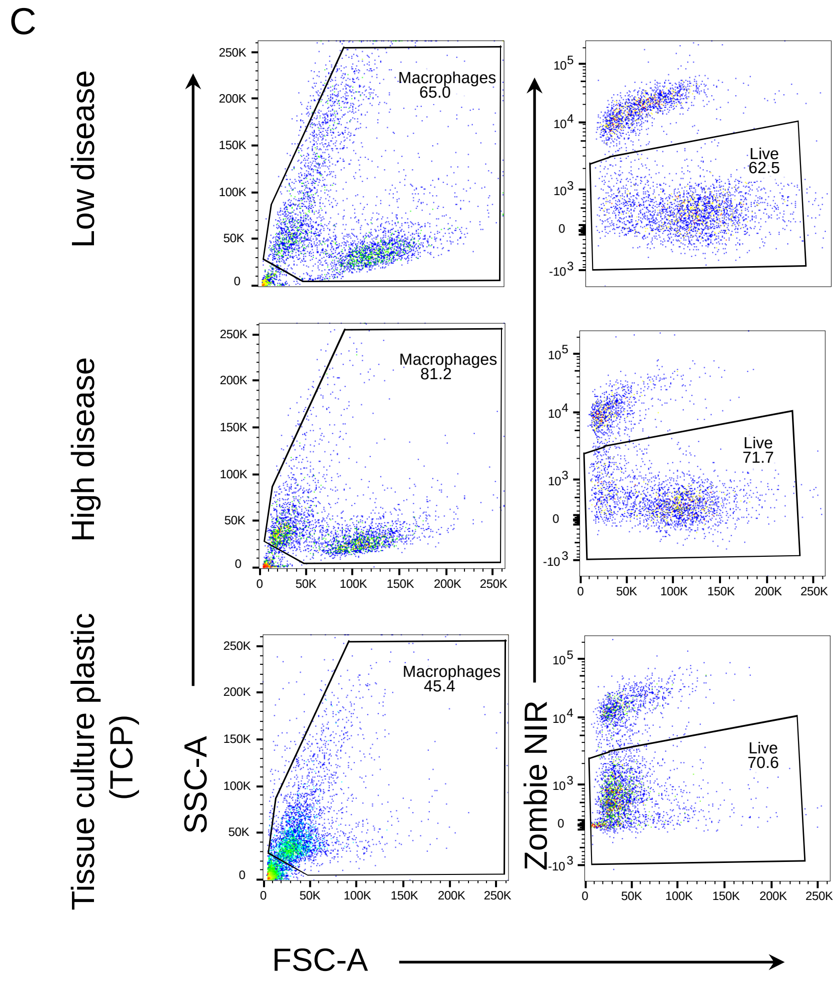

Supplementary Figure 11. C) Representative pseudocolor plots of macrophages recovered from decellularized tissue or tissue culture plastic (TCP) cultures on day 14. Low disease: G145; high disease: G343.

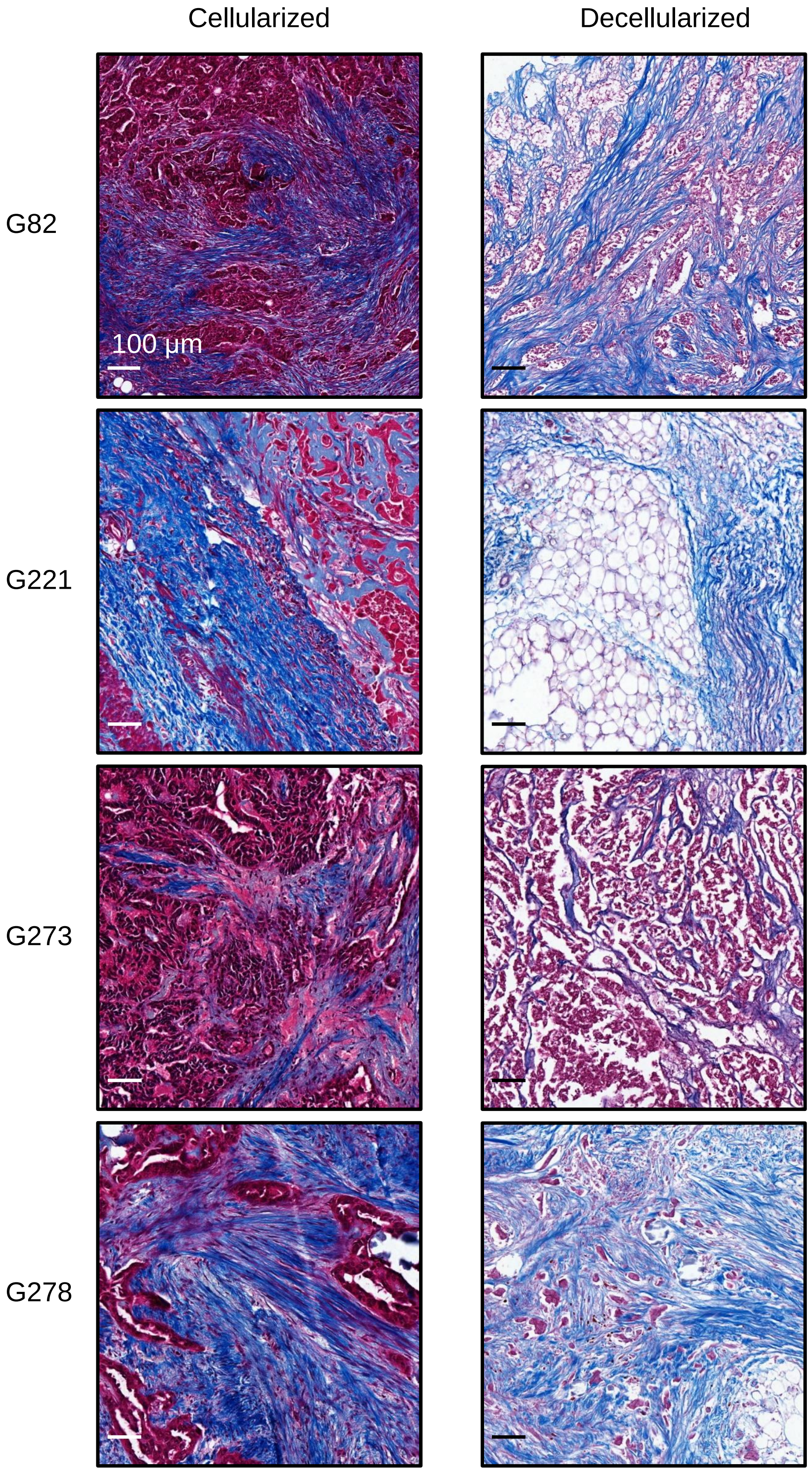

Supplementary Figure 12. Representative images of cellularized and decellularized tissue. Stained by Masson's trichrome stain (N = 4). Scale bar = 100µm.

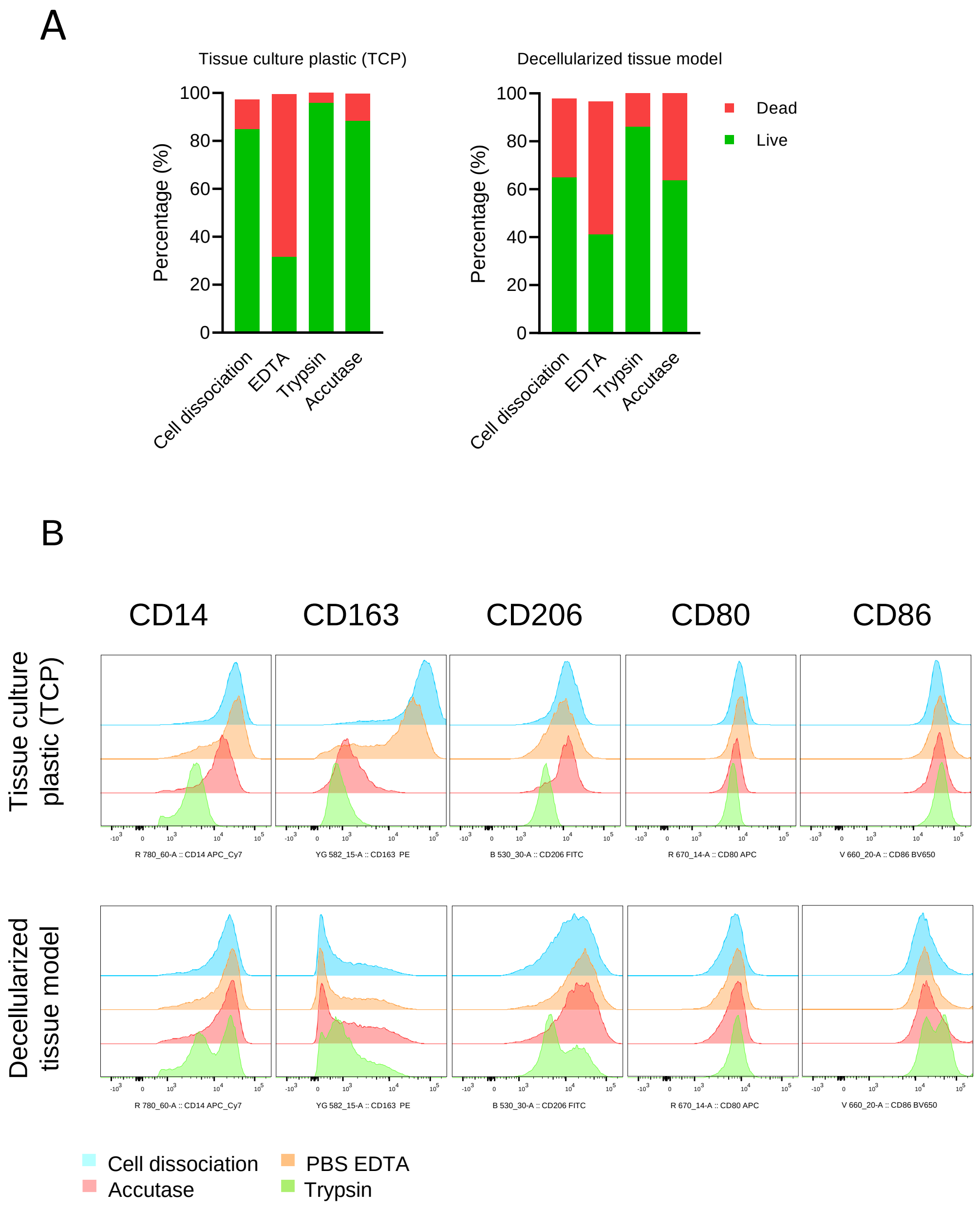

Supplementary Figure 13. Optimisation of macrophage cell recovery for flow cytometry analysis. A) Live/ dead analysis of macrophages cultured on general tissue culture plastic (TCP) and in the decellularized tissue model, and the four detachment methods used to recover cells (Cell dissociation buffer, PBS supplemented with 5mM EDTA, PBS supplemented with 0.5%/ 0.2% Trypsin/ EDTA, and Accutase buffer). **B)** Histogram overlays comparing marker expression of macrophages between collection methods and between MO and the decellularized tissue model cultures. Green – trypsin, Red – accutase, Orange – PBS EDTA, and Blue – cell dissociation buffer. N = 2.

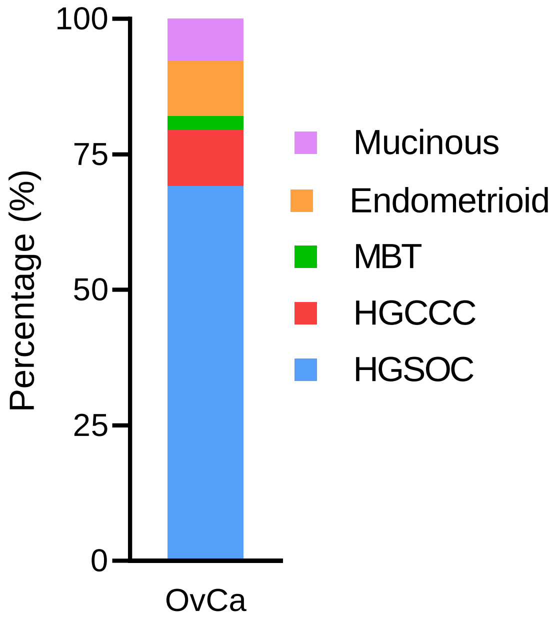

Supplementary Figure 14. 39 Ovarian cancer (OvCa) patients were used to build the omentum tissue library. HGSOC: high grade serous ovarian cancer, HGCCC: high grade clear cell cancer, MBT: malignant Brenner tumor.

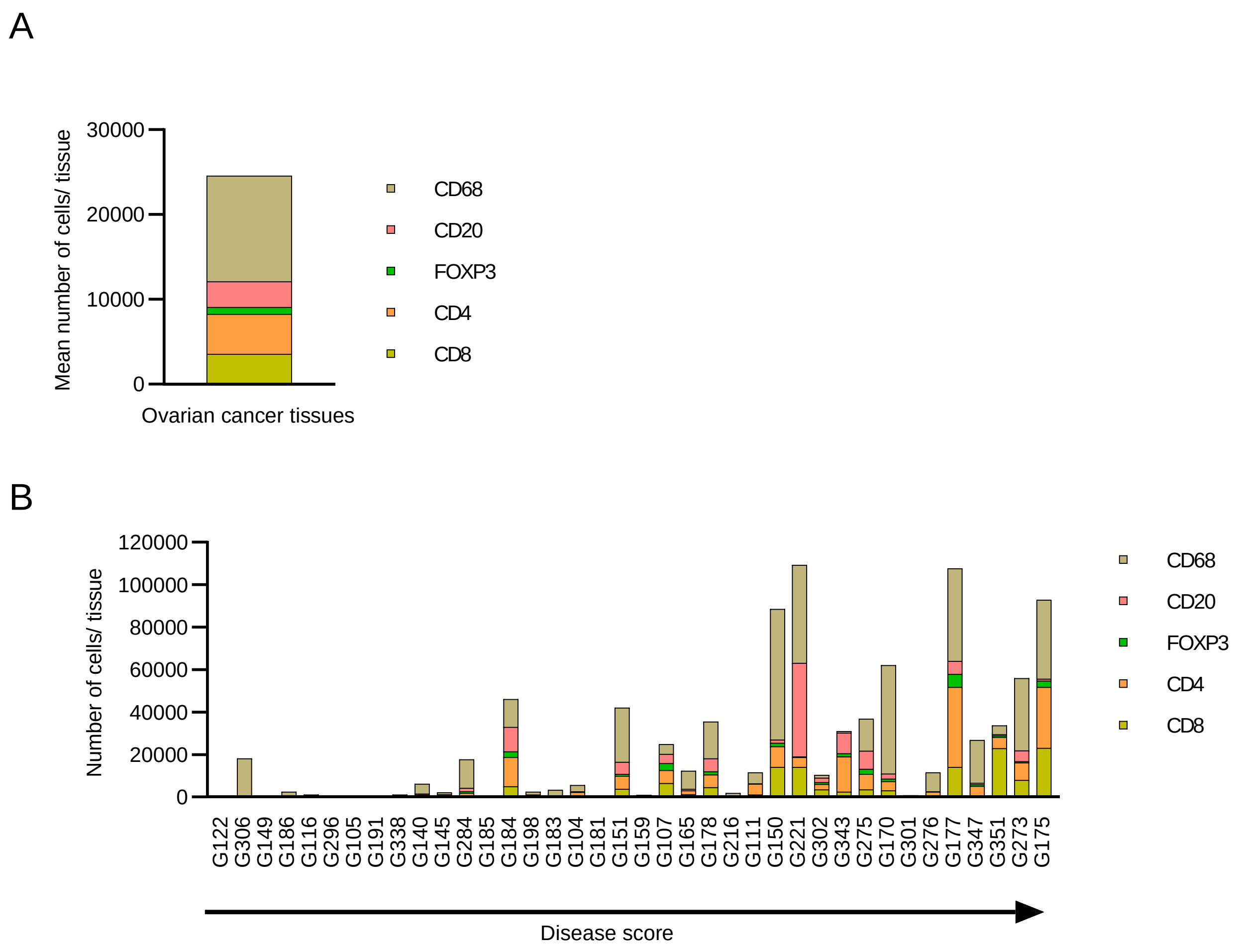

Supplementary Figure 15. Overview of the immune landscape. A) Barplot of mean number of cells/tissue for IHC staining for CD68, CD20, FOXP3, CD4 and CD8 immune cells across all ovarian cancer tissues. N = 38. **B)** Barplot of number of cells/tissue for IHC staining for CD68, CD20, FOXP3, CD4 and CD8 immune cells for each individual ovarian cancer tissue ranked by disease score.

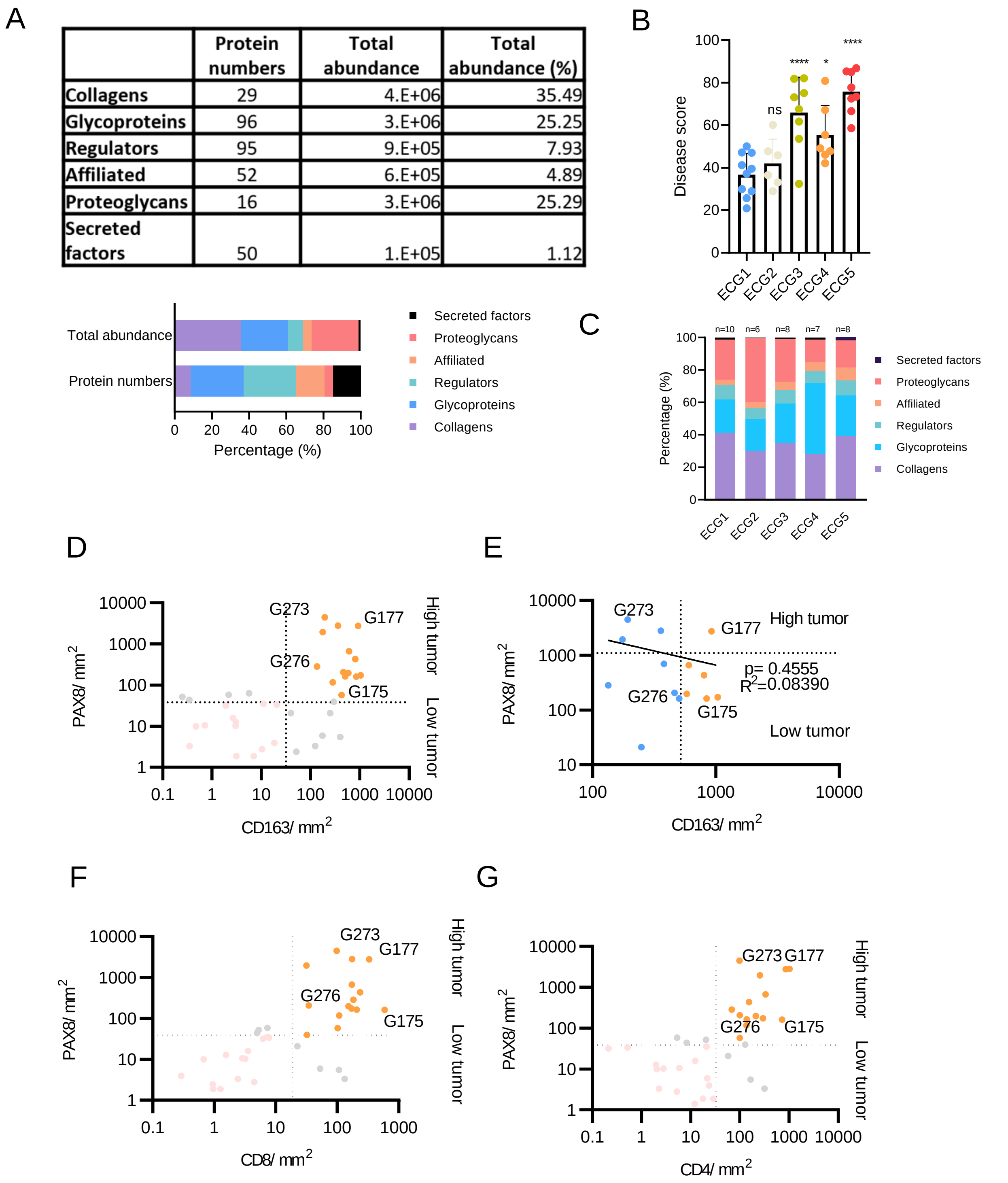

	Protein numbers	Total abundance	Total abundance (%)
Collagens	29	4.E+06	35.49
Glycoproteins	96	3.E+06	25.25
Regulators	95	9.E+05	7.93
Affiliated	52	6.E+05	4.89
Proteoglycans	16	3.E+06	25.29
Secreted factors	50	1.E+05	1.12

Supplementary Figure 16. Matrisome heterogeneity of ovarian cancer omentum. A) Protein numbers and total abundances of matrisome categories summed from proteomic enrichment values. Plotted values corresponding to Table A as percentage. N = 39. **B)** Disease score values between ECGs. Data are presented as mean values +/- SD. One way ANOVA with Dunnett's post-hoc test, significance between each group is presented as ****$p < 0.0001$ and *$p = 0.0167$. ECG1 N = 10 samples; ECG2 N = 6 samples; ECG3 N = 8 samples; ECG4 N = 7 samples; ECG5 N = 8 samples. **C)** Matrisome categories between ECGs. **D)** Scatter plot of $PAX8^+$ cells against $CD163^+$ cells across OvCa samples. Geometric mean of $PAX8^+$ and $CD163^+$ values were calculated and indicated by dashed line and defined samples by low or high macrophage counts in context to total samples. **E)** OvCa high disease only samples. Geometric mean $PAX8^+$ and $CD163^+$ values were calculated and indicated by dashed line in context to high macrophage samples only (from Supplemental Figure 13F). Least squares regression line. $R^2 = 0.4555$, $p = 0.08390$. **F)** Scatter plot of $PAX8^+$ cells against $CD8^+$ cells across OvCa samples. Geometric mean of $PAX8^+$ and $CD163^+$ values were calculated and indicated by dashed line and defined samples by low or high macrophage counts in context to total samples. **G)** Scatter plot of $PAX8^+$ cells against $CD4^+$ cells across OvCa samples. Geometric mean of $PAX8^+$ and $CD163^+$ values were calculated and indicated by dashed line and defined samples by low or high macrophage counts in context to total samples.

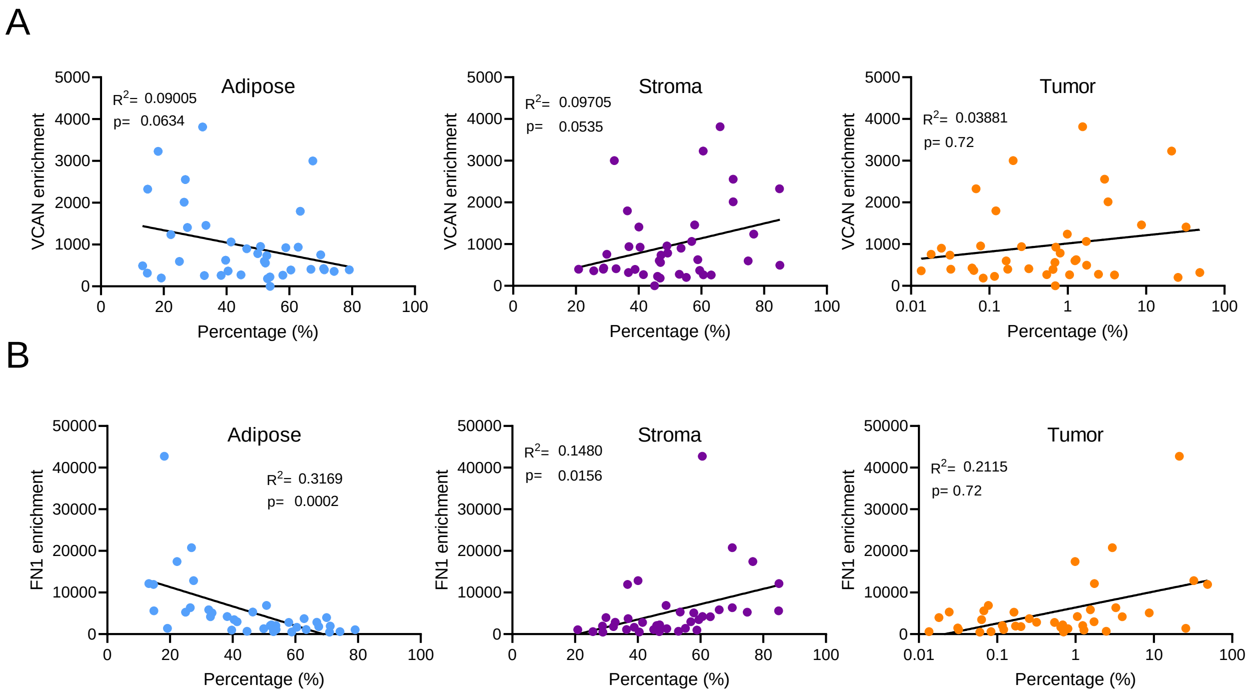

Supplementary Figure 17. Versican and fibronectin are heterogeneous matrisome proteins which associate with increased stroma. A) Versican and **B)** fibronectin mass ratio values (enrichment) associated with percentage (%) of adipose, stroma and tumour as defined by Definiens® digital analysis. Simple linear regression. N = 38.

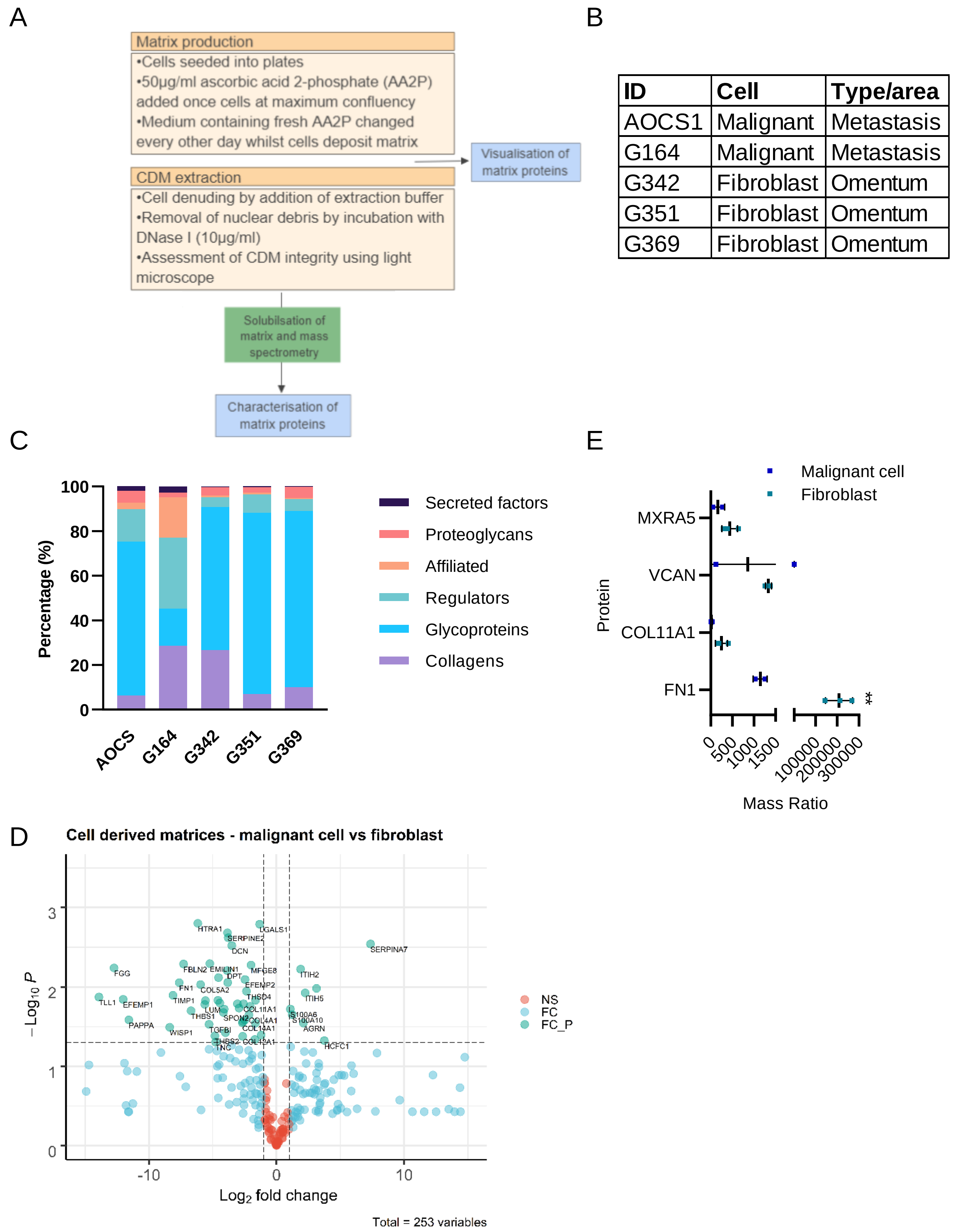

ID	Cell	Type/area
AOCS1	Malignant	Metastasis
G164	Malignant	Metastasis
G342	Fibroblast	Omentum
G351	Fibroblast	Omentum
G369	Fibroblast	Omentum

Supplementary Figure 18. Matrisome molecules associated with M0 macrophages are enriched in cell-derived matrices (CDMs) from patient-derived omental fibroblasts compared to HGSOC metastatic malignant cells. A) Schematic summarising the culture methods involved in the generation of CDMs **B)** Table of *in vitro* cultured patient-derived cell types **C)** Malignant and fibroblast CDMs classified into matrisome categories **D)** Volcano plot of p-value and log2FC for matrisome proteins. Red = non-significant with log2 fold change <1 or >-1(NS), blue = non-significant with log2 fold change >1 or <-1 (FC), green = significant with log2 fold change >1 or <-1 (FC_P). T tests for differential expression. **E)** Averaged mass ratio for matrisome protein expression associated with M0 macrophages in malignant cells and fibroblasts. Data are presented as mean values +/- SD. Mixed-effects analysis with Sidak's multiple comparisons test. ** p-value = 0.0022. N = 3 fibroblast samples, N = 2 malignant cell samples.

A

ID	Type	Disease score
G164	High Disease	81.96013
G302	High Disease	67.54765
G276	High Disease	77.65805
G150	High Disease	66.59231
G122	Low Disease	20.91347
G306	Low Disease	25.73339
G198	Low Disease	46.95865
G145	Low Disease	41.16155

B

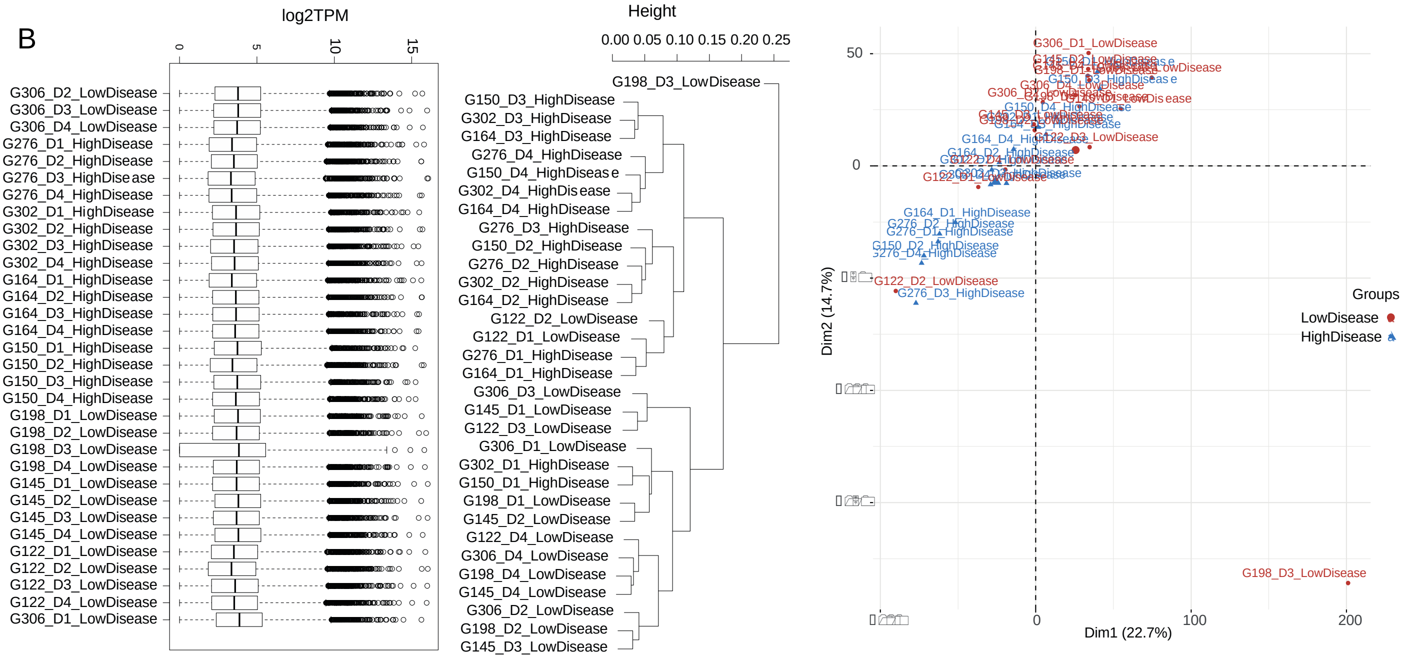

C

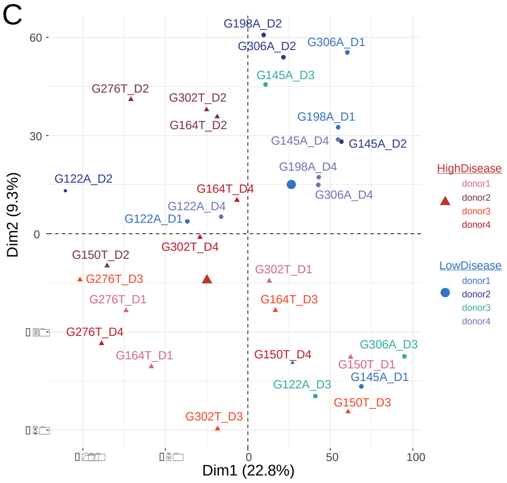

D

Theshold	Number of DE genes
protein-coding adjp =< 0.05 logFC >= 0	1839
protein-coding adjp =< 0.05 logFC <= 0	1774
protein-coding adjp =< 0.05 logFC >= 1	261
protein-coding adjp =< 0.05 logFC <= -1	198

E

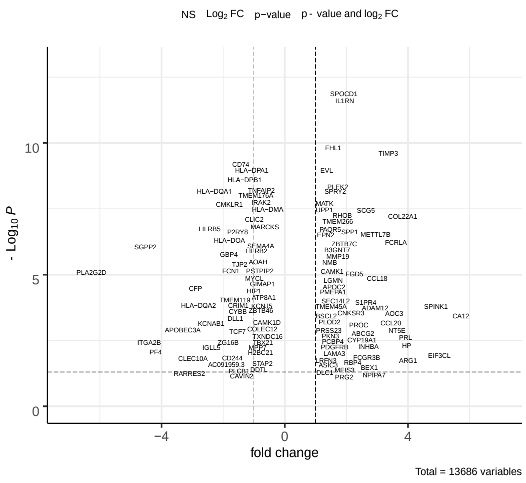

Supplementary Figure 19. High disease ECM alters the macrophage transcriptome. **A)** List of decellularized tissues used for RNAseq macrophage cultures. N = 8. **B)** Exploratory boxplot for sample quality, unsupervised cluster analysis and PCA revealed G198_D3 poor quality. Boxplots illustrate median (centre of the box) with the upper (Q3: 75th percentile) and lower (Q1: 25th percentile) quartiles (ends of the box); The whiskers correspond to Q3 + 1.5 x Interquartile Range (IQR) to Q1 - 1.5 x IQR; Dots beyond the whiskers show potential outliers. N = 32 samples, 4 blood donors, x4 high disease samples, x4 low disease samples. **C)** PCA using all genes. **D)** Table of number of differentially expressed (DE) genes identified using the lm model of the limma R package and voom normalization with a ~ECM.type+donor model design. LogFC > 0 denotes up in high disease, logFC < 0 denotes down in high disease. More stringent analysis used fold change greater than 2 (logFC ≥ 1 or logFC ≤ -1). **E)** Volcano plot of p-values and Log_2FC of high versus low disease tissue cultured macrophages. T tests for differential expression.

A

B

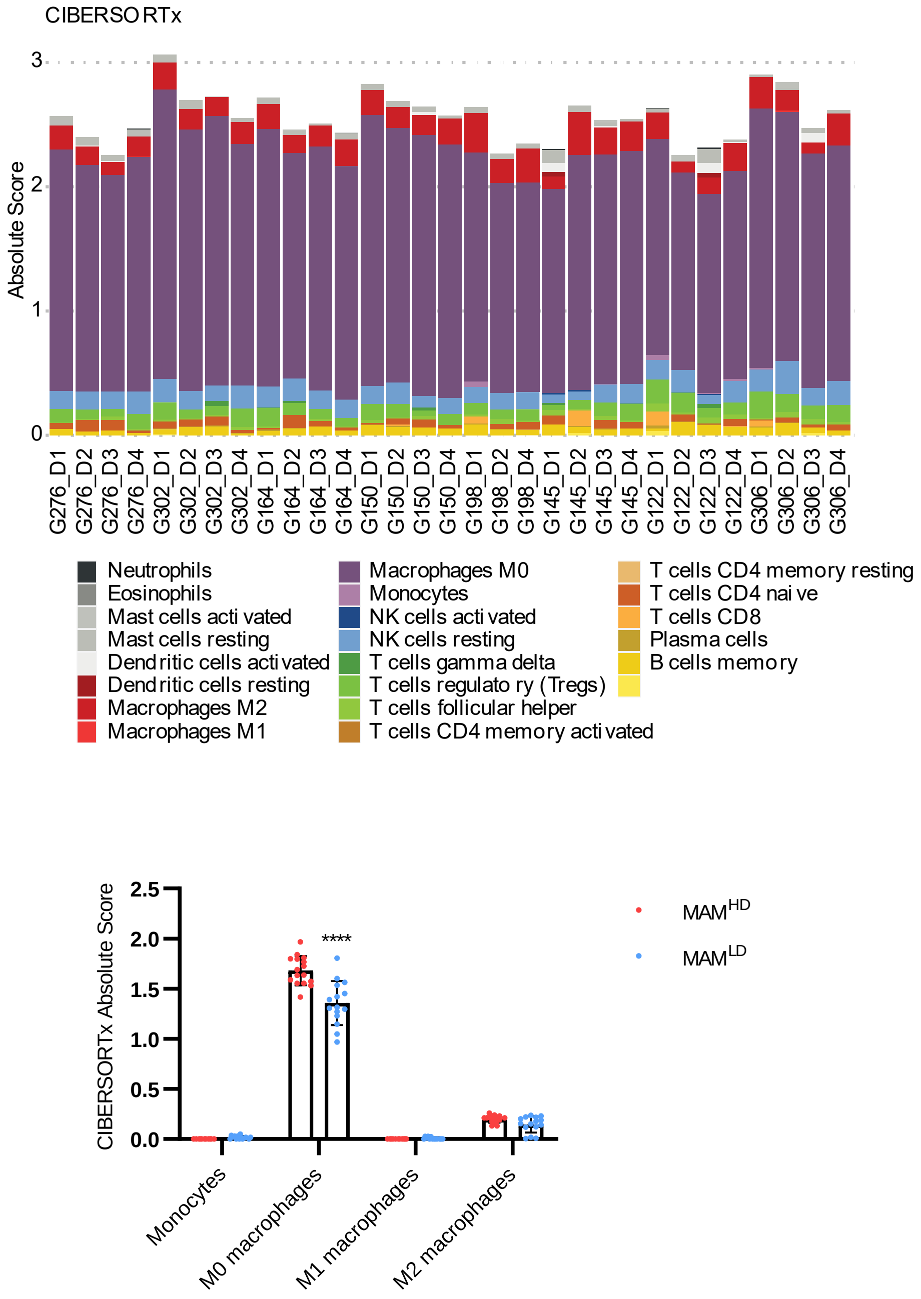

Supplementary Figure 20. High disease extracellular matrix-educated macrophages share signature with M0 macrophages. A) Stacked bar chart of immune cell predictions by CIBERSORTx grouped by monocytes cultured on high or low disease decellularized tissue. Cell color codes are presented in a key below with color matching across methods. **B)** Barplot of all macrophage subsets and monocytes identified in high and low disease MAM populations from CIBERSORTx analysis on DE genes. Data are presented as mean values +/- SD. Two-way ANOVA with Sidak's multiple comparison test. ****$p < 0.0001$. N = 16 HD MAM samples, N = 15 LD MAM samples.

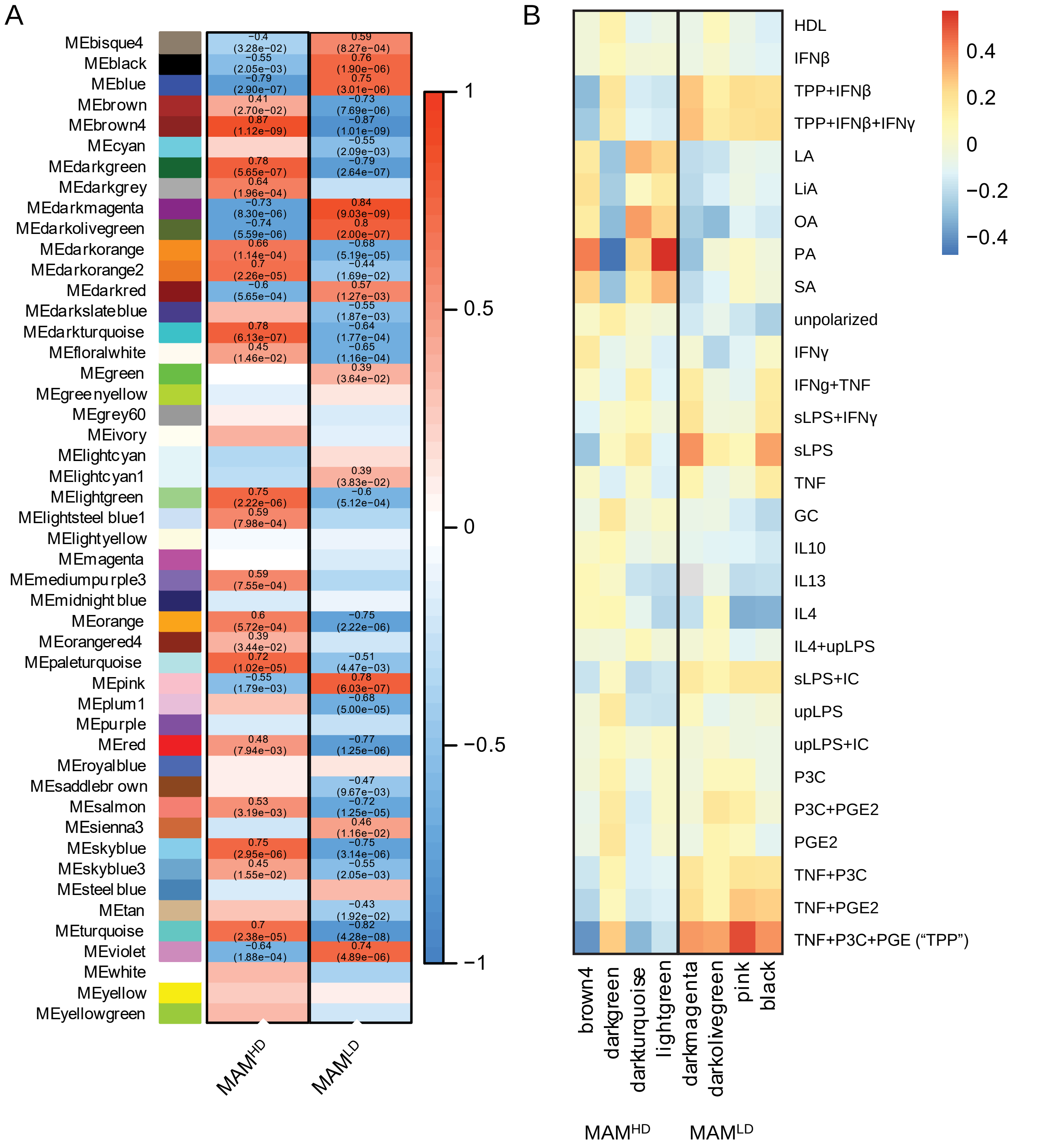

Supplementary Figure 21. High and low disease MAMs share similar features with macrophages activated by fatty acid stimuli and LPS stimuli, respectively. A) Heatmap showing correlation between colored module eigengenes (ME) taken from Xue et al., 2014 and genes significantly up-regulated in high disease MAMs (MAM^{HD}) compared to low disease MAMs (MAM^{LD}). Each module of the 49 modules has been ascribed a unique colour and colours name. Red indicates a positive correlation, blue indicates a negative correlation. Significant p-values are indicated. **B)** Correlation of top 4 high disease MAM and low disease MAM ME patterns with biological stimuli.

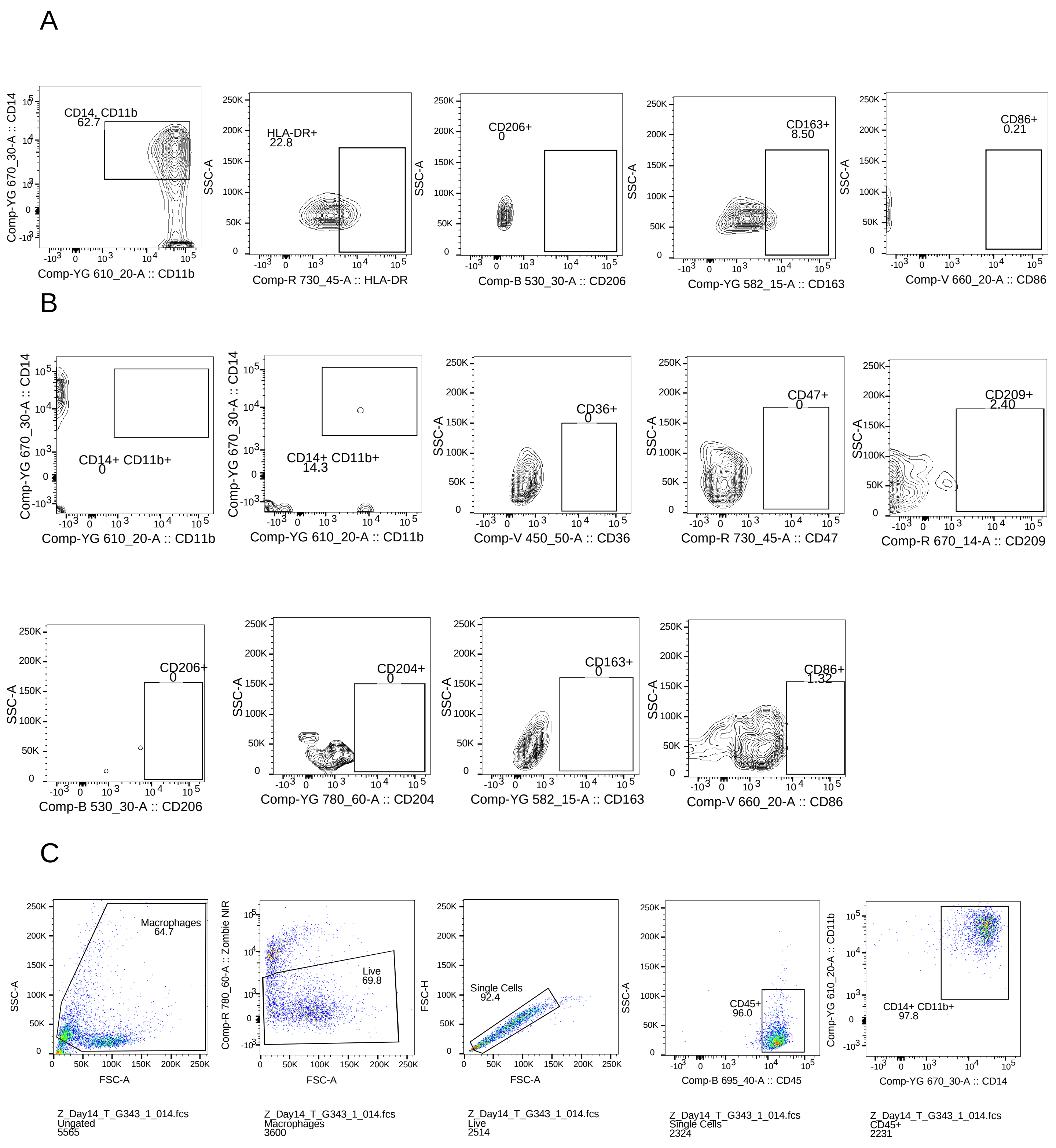

Supplementary Figure 22. Day 0 healthy human PBMC derived monocytes used for decellularized tissue culture. A) PBMC derived monocytes isolated at day 0 from healthy human blood donors were analyzed by flow cytometry for transmembrane marker expression. Representative cell contour plots of day 0 monocytes marker expression. Same donor monocytes were seeded onto decellularized tissues and later collected and analyzed by flow cytometry and RNAseq after 14 days of culture (Figure 4-5). **B)** Fluorescence minus one (FMO) controls. **C)** Gating strategy used to select macrophages collected from decellularized tissues.

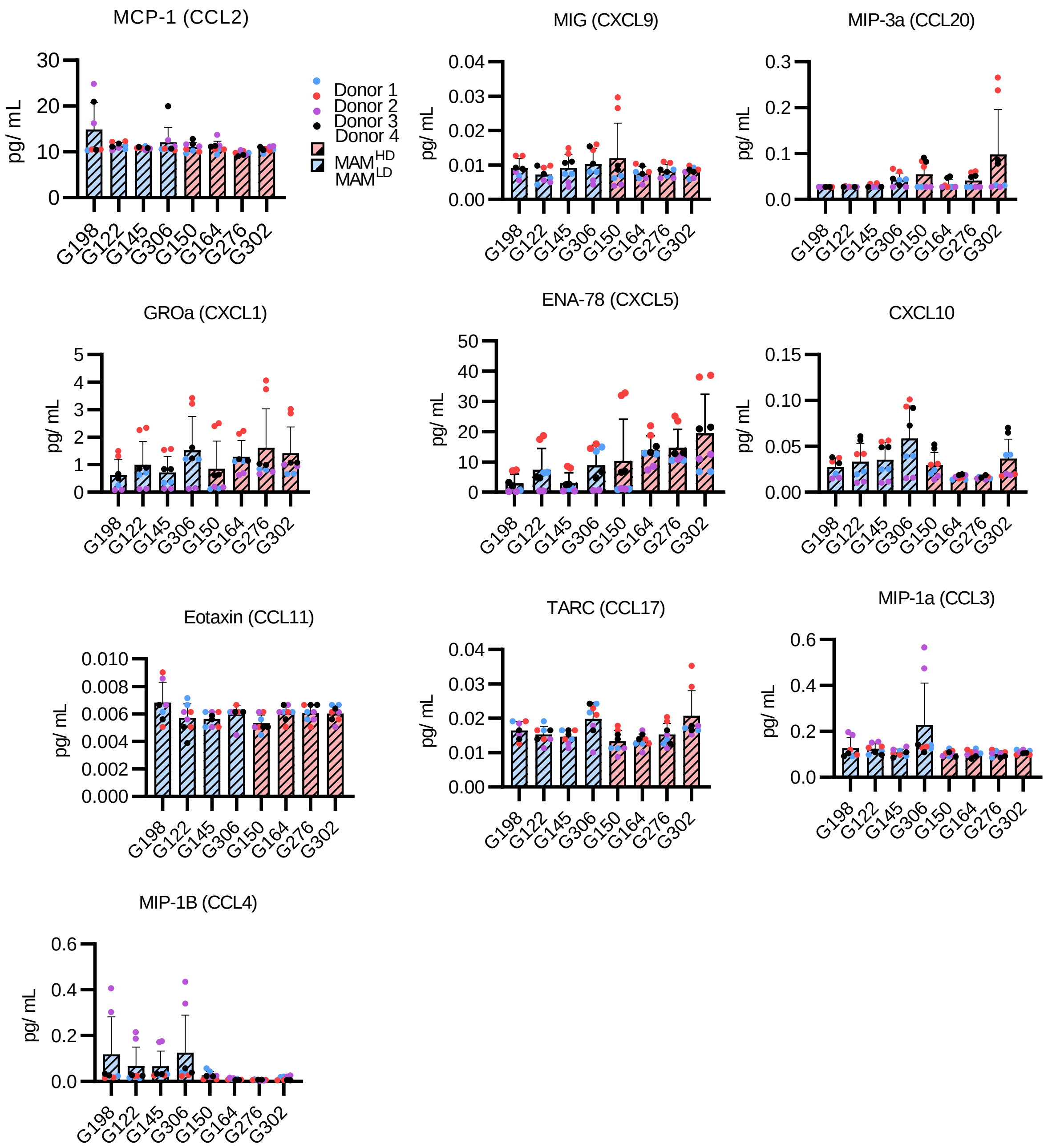

Supplementary Figure 23. High disease ECM alters the macrophage secreted chemokines. LEGENDplex™ quantification (pg/mL) of chemokine levels in macrophage supernatants from high and low disease ECM cultured macrophages. Data are presented as mean values +/- SD. N = 4 tissues each, x4 donors per tissue. Duplicates used for each donor.

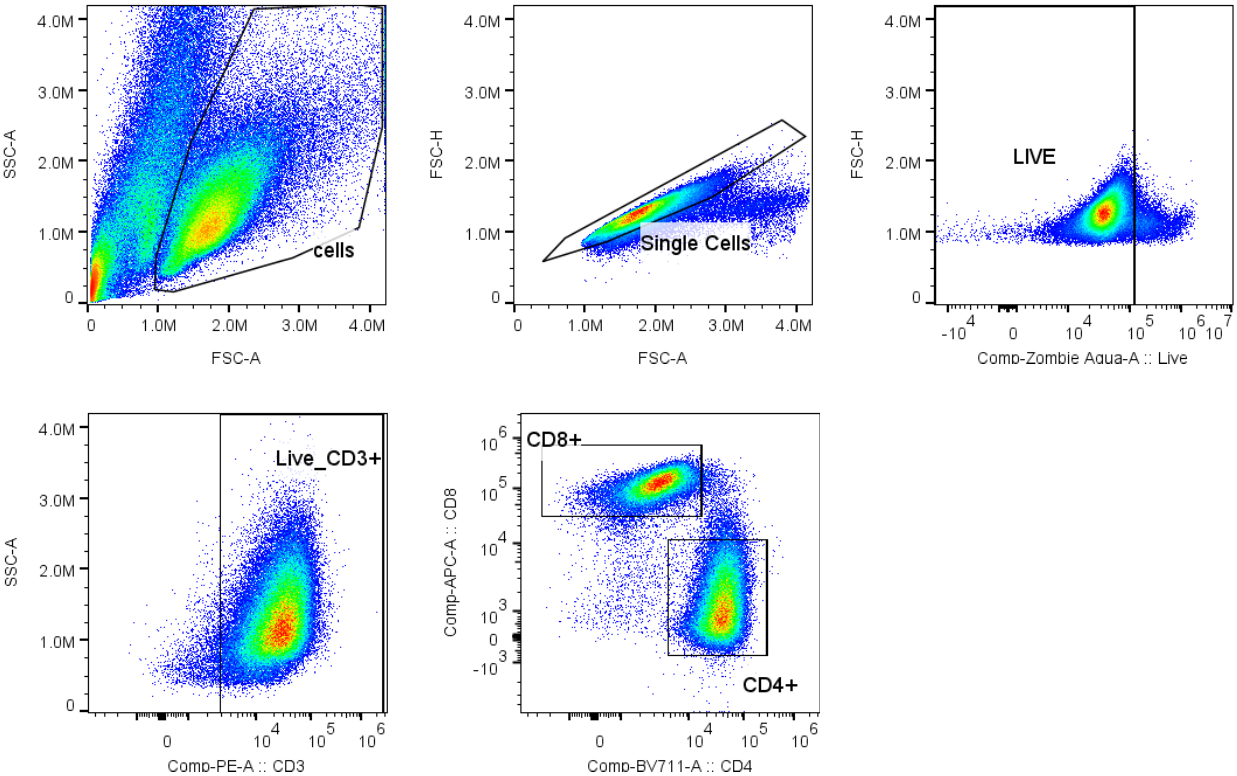

Supplementary Figure 24. Flow gating strategy for T cell flow cytometry assay. CD3+ T cells as assessed using flow cytometry after 5 days culture alone or co-culture with high or low disease MAMs conditioned media (Figure 6). N = 4.

Supplementary Methods

Data collection. The raw HGSOC count per million (CPM) RNA-seq dataset (GSE71340) collected in a previous study[1], was downloaded from the NCBI Gene Expression Omnibus (GEO) database[70] and protein mass ratios (PXD004060[1]) from the PRIDE database[69]. Corresponding HGSOC cellularity data, obtained by manually counting six major leukocyte subtypes was obtained from the CanBuild site (canbuild.org.uk[1]). While 36 patient samples were initially obtained, only 32 samples have all 3 corresponding datasets.

Deconvolution of bulk RNA-seq profiles. Log2 base RNA-seq data was delogged and processed to fit the input requirements of each computational method. CIBERSORT and CIBERSORTx estimations were performed against the standard LM22 signature gene file in absolute mode with 100 permutations and quantile normalisation disabled. For CIBERSORTx, impute cell fractions analysis module was selected in custom mode, with B-mode batch correction applied using LM22 source GEP. xCell analysis was performed using the N = 64 gene signature and the RNA-seq box ticked.

Merging cell subtype estimates for correlation to IHC marker cell counts. We evaluated the performance of three deconvolution methods, CIBERSORT, CIBERSORTx, and xCell, by Spearman correlation between the cell type proportions computed by the different deconvolution methods and known compositions from IHC. From the default cell type estimations by CIBERSORT, CIBERSORTx and xCell, we identified all cell types that express each of the six immune markers for which cell count data was available (CD3, CD4, CD8, CD45RO, FOXP3, CD68). CD3 cell scores are calculated as the sum of CD8 T cells, CD4 naïve T cells, $\gamma\delta$ T cells, CD4 memory T cells and follicular helper T cells (CIBERSORT and CIBERSORTx only) abundances; CD4 cell scores the sum of CD4 naïve T cells, T regs, CD4 memory cells, follicular helper T cells (CIBERSORT and CIBERSORTx only), $\gamma\delta$ T cells, in addition to CD4 Tem and CD4 Tcm for xCell only; CD8 cell scores as just CD8 T cells for CIBERSORT and CIBERSORTx, in addition to CD8 Tcm, CD8 Tem and CD8 naïve T cells for xCell. CD68 cell scores are calculated as the sum of monocytes, macrophages M0 (CIBERSORT and CIBERSORTx only) or macrophages (xCell only), M1 and M2, and dendritic cells (no immature DC); CD45RO just CD4 memory T cells and FOXP3 just Tregs. Spearman's correlation analysis between the marker cell count data and the marker cell estimation scores was then performed (Supplementary Figure 1D and E). A limitation of our evaluation of the bulk deconvolution methods is that immune cell type proportions were assessed using IHC for only six markers: $CD3^+$, $CD4^+$, $CD8^+$, $CD68^+$, $CD45RO^+$ and $FOXP3^+$. As 22 immune cell types are computed by CIBERSORT and CIBERSORTx, and 34 immune cell types are computed by xCell, computed immune cell types were combined to assess

their correlation against the IHC cell counts (e.g. xCell $CD8^+$ naïve T cells, $CD8^+$ Tcm and $CD8^+$ Tem computed values were combined to correlate against $CD8^+$ IHC immune cell counts). The gold standard to evaluate bulk deconvolution would be to compare against cell type proportions measured using single cell RNA seq or high resolution multiparameter flow cytometry which has not been possible to perform here but has been performed comprehensively in the literature[19,20,22].

Clinical outcome analysis. Delogged HGSOC RNA-seq data was input to estimate the tumor progression within the cancer immunity cycle, using the tracking tumor immunophenotype (TIP) meta-server[31]. Samples were separated into the top 12 high and bottom 12 low expressing samples. Kaplan-Meier plots, hazard ratios and log ranked p significance values were generated using gene expression omnibus (GEO), European genome archive (EGA) and the cancer genome atlas (TCGA) RNA-seq databases, via the KM plotter online meta-analysis tool[71].

RNA isolation and sequencing. Total RNA was extracted from macrophage cultures on decellularized tissue using RLT buffer (Qiagen) and rigorously vortexed. Samples were processed using the RNeasy Micro Kit (Qiagen) following manufacturer's instructions. RNA quality and integrity assessments were performed at Oxford Genomics. RNA sequencing was performed at Oxford Genomics. Material was quantified using RiboGreen (Invitrogen) on the FLUOstar OPTIMA plate reader (BMG Labtech) and the size profile and integrity analysed on the 2200 or 4200 TapeStation (Agilent, RNA ScreenTape). RIN estimates (where available) were between 7 and 9.7. Input material was normalised to 10 ng (or maximum mass available) prior to library preparation. Polyadenylated transcript enrichment and strand specific library preparation was completed using NEBNext Ultra II mRNA kit (NEB) following manufacturer's instructions. Libraries were amplified (18 cycles) on a Tetrad (Bio-Rad) using in-house unique dual indexing primers (based on DOI: 10.1186/1472-6750-13-104). Individual libraries were normalised using Qubit, and the size profile was analysed on the 2200 or 4200 TapeStation. Individual libraries were normalised and pooled together accordingly. The pooled library was diluted to ~10 nM for storage. The 10 nM library was denatured and further diluted prior to loading on the sequencer. Paired end sequencing was performed using a NovaSeq6000 platform (Illumina, NovaSeq 6000 S2/S4 reagent kit v1.5, 300 cycles), generating a raw read count of >23 million reads per sample.

Cytokine and chemokine analysis. Cytokine and chemokines were assayed using LEGENDPLEX™ platform according to manufacturer's instructions. The human proinflammatory chemokine panel 1 kit (cat: 741081) was used to quantitatively measure the levels of 12 human chemokines from macrophage conditioned media. Chemokines included CXCL8, CXCL10, CCL11, CCL17, MCP-1, CCL3, CXCL9, CXCL5,

CCL20, CXCL1, CXCL11, CCL4. A total of 32 conditions were analysed in duplicates and followed manufacturer's instructions. The Biolegend cloud-based data analysis software suite was used to analyse flow cytometry data. Top standards were set based on manufacturers guide for that specific lot number kit (B326082) and were: CXCL8 (21ng/mL), CXCL10 (11ng/mL), CCL11 (12ng/mL), CCL17 (10ng/mL), MCP-1 (13ng/mL), CCL3 (32ng/mL), CXCL9 (4ng/mL), CXCL5 (6ng/mL), CCL20 (2ng/mL), CXCL1 (4ng/mL), CXCL11 (4ng/mL), CCL4 (3ng/mL).

Histochemical analysis. Frozen tissues were fixed in in 4 % paraformaldehyde (PFA) and cryosectioned to 8-10 µm slices. All tissue sections were scanned using a 3DHISTECH Panoramic 250 digital slide scanner (3DHISTECH, Hungary) and the resulting scans were analysed using Definiens software (Definiens AG, Germany). Disease scores were determined firstly by manually defining regions of interest in the tissue that represented tumor (PAX8), stroma, and fat (adipocytes) and then training the software to recognize these regions of interest. Disease score was expressed as a percentage of the whole tissue area that contained tumor and/or stroma (Figure 2B-D). Paraffin embedded tissues were submerged in xylene and then a series of ethanol washes of decreasing concentration for 2 x 2 min each (100 %, 90 %, 70 %, and 50 %). Antigen retrieval was performed for 10 min using vector antigen unmasking buffer and a pressure cooker. Tissue sections were then washed with DAKO wash buffer followed by application of H2O2 for 5 min. Blocking was performed using 5 % BSA for 20 min at RT followed by incubation with primary antibody in biogenex antibody diluent for 30 min. After 3 x washes, biogenex super enhancer was added for 20 min and then washed off before addition of biogenex ss label poly-HRP for 30 min. Tissues were washed three times before addition of DAB chromagen for 3 min followed by washing to stop further DAB development. Tissues were counterstained with haematoxylin followed by washing with H2O and ethanol solutions of increasing concentration for 2 min each (50 %, 70 %, 90 %, 100 %) and then 2 x xylene. Samples were then mounted and scanned using the 3DHISTECH Panoramic digital slide scanner. Immune cells were counted using QuPath.

Matrix staining. Immunohistochemical staining for ECM proteins was performed on 4 µm slides of FFPE human omentum tissue as described above.

Antibodies. The following antibodies were used for immunohistochemical analyses: anti-PAX8 (clone BC12, ab124445; 1:1000; Abcam), anti-FOXP3 (clone eBio7979 (221D/D3), 14-7979-82; 1:1500; Invitrogen, UK); anti-CD4 (clone 4B12, M7310; 1:300; Dako), anti-CD8 (clone C8/144B, ab75129; 1:500; Abcam), anti-CD68 (clone PG-M1; GA613; 1:12000; Dako), anti-CD163 (clone 10D6, MA5-11458; 1:1000; Thermo Fisher), anti-CD20cy (clone L26; M0755; 1:400; Dako), anti-VCAN (polyclonal, Ab202906; 1:200;

Abcam), anti-COL1A1 (polyclonal, HPA011795; 1:500; Sigma Aldrich), anti-FN1 (polyclonal, Ab23750; 1:500; Abcam), anti-CTSB (ab125067; 1:50; Abcam), anti-CS (clone CS-56, Ab11570; 1:600; Abcam).

Flow cytometry analysis. Macrophages were initially washed using PBS and then stained with 1:1000 Zombie™ NIR (1:1000, Cat: 423106, Biolegend) in PBS supplemented with Fc receptor and monocyte blocker (Biolegend) for 20 minutes at RT. Cells were then washed and stained in 50µL total volume PBS, 2% BSA, 2mM EDTA (FACS buffer) using an antibody master mix for 20 minutes at 4°C. Subsequent stained cells were washed in FACS buffer and analysed. Monocyte derived macrophage staining panel used: CD206 (FITC, 1:100, Cat: 321104, Biolegend), CD45 (PerCP, 1:100, Cat: 368506, Biolegend), CD209 (APC, 1:100, Cat: 330107, Biolegend), CD47 (AF700, 1:100, Cat: 323125, Biolegend), CD36 (BV421, 1:100, Cat: 336229, Biolegend), CD38 (BV605, 1:100, Cat: 356641, Biolegend), CD86 (BV650, 1:100, Cat: 305428, Biolegend), CD163 (PE, 1:100, Cat: 333606, Biolegend), CD11b (AF594, 1:100, Cat: 301340, Biolegend), CD14 (PE-Cy5, 1:100, Cat: 15-0149-42, Invitrogen), and CD204 (PE-Cy7, 1:100, Cat: 371907, Biolegend). Cytofluorimetric analysis was performed using a 4-laser Fortessa flow cytometer (BD). For macrophages 2,000 live events were collected and selected by Zombie NIR gating strategy. Unstained, Zombie™ stained cells spiked with unstained cells and fluorescence minus one (FMO) for each antibody in the panel were used as controls to set up cell gating strategies. Compensation control beads (UltraComp eBeads™ compensation beads, Thermofisher; 01-2222-42) were made for each antibody marker and were used to calculate the overlap of fluorescence between laser channels and automatically calculated and applied to each full stain sample. This panel of markers was validated using a titration of antibodies at various concentrations and staining monocytes at day 0 and differentiated macrophages at day 7 and 14. Data was analysed using Flowjo.

T Cell flow cytometry. T cells were harvested and preincubated with the anti-human Fc-Receptor binding inhibitor (Invitrogen) following the manufacturer's instruction. Cells were then stained for 30 minutes on ice using monoclonal antibodies to the following human proteins: CD223-FITC (1:50, 3DS223H, ThermoFisher), CD8-APC (1:100, SK1, Biolegend), CD366-APCCy7 (1:50, F38-2E2, Biolegend), CD4-BV711 (1:100, SK3, BD Bioscience), CD3-PE (1:100, UCHT1, Biolegend) and CD279-PECy7 (1:20, EH12.1, BD Bioscience). Viability was tested using the Zombie Aqua Fixable dye (Biolegend). Samples were acquired on LSRFortessa I (Becton Dickinson) and analysed using FlowJo v10.7 (BD FlowJo LLC). Flow gating strategy provided in Supplementary Figure 24.

Disease score quantification. The level of disease was calculated via IHC analysis of whole tissue sections as a disease score (Supplementary Figure 9B-D). The disease score was devised by a scoring system that

quantified the sum of the area of tumor cells (in this case we used PAX8 as marker of ovarian cancer cells) and tissue stroma (Supplementary Figure 9B-C). The tissue stroma was the area of desmoplasia (or tumor ECM) resulting from the invasive tumor within the tissue. Whole tissue sections were analyzed using Definiens® digital image software, which calculated the percentage of the tumor, stroma and adipose content within the tissues (Supplementary Figure 9C-D). The 39 samples were clinically graded as stage III-IV and were collected predominantly from patients with HGSOC, but also patients with high grade clear cell carcinoma, malignant Brenner tumor, Borderline serous cystadenoma, benign fibroma, benign mucinous cystadenoma, sero-mucinous borderline tumor and mucinous adenocarcinoma (Supplementary Table 5 and Supplementary Figure 13). QuPath was used to quantify $PAX8^+$ cells (Supplementary Figure 9E) and $PAX8^+$ cell counts positively correlated with the disease score (Supplementary Figure 9F), indicating the quantity of tumor cells associated with the level of ECM remodeling. The clinical staging of samples did not correlate with the tissue disease score. We used the disease score to account for differences between tissues that may result from the level of disease present.
