# Supplementary material for: Extracellular matrix educates an immunoregulatory tumor macrophage phenotype found in ovarian cancer metastasis
Source: Nat Commun. 2023 May 15;14:2514. doi: 10.1038/s41467-023-38093-5 (PMC10185550; doi:10.1038/s41467-023-38093-5)
Supplement: Supplementary file 3 — Reporting Summary [file 41467_2023_38093_MOESM3_ESM.pdf]

nature portfolio

Corresponding author(s): Oliver M. T. Pearce

Last updated by author(s): 17/03/23

Reporting Summary

Nature Portfolio wishes to improve the reproducibility of the work that we publish. This form provides structure for consistency and transparency in reporting. For further information on Nature Portfolio policies, see our Editorial Policies and the Editorial Policy Checklist.

Statistics

For all statistical analyses, confirm that the following items are present in the figure legend, table legend, main text, or Methods section.

n/a	Confirmed	
☐	☒	The exact sample size (*n*) for each experimental group/condition, given as a discrete number and unit of measurement
☐	☒	A statement on whether measurements were taken from distinct samples or whether the same sample was measured repeatedly
☐	☒	The statistical test(s) used AND whether they are one- or two-sided<br>*Only common tests should be described solely by name; describe more complex techniques in the Methods section.*
☒	☐	A description of all covariates tested
☒	☐	A description of any assumptions or corrections, such as tests of normality and adjustment for multiple comparisons
☐	☒	A full description of the statistical parameters including central tendency (e.g. means) or other basic estimates (e.g. regression coefficient) AND variation (e.g. standard deviation) or associated estimates of uncertainty (e.g. confidence intervals)
☐	☒	For null hypothesis testing, the test statistic (e.g. *F*, *t*, *r*) with confidence intervals, effect sizes, degrees of freedom and *P* value noted<br>*Give P values as exact values whenever suitable.*
☒	☐	For Bayesian analysis, information on the choice of priors and Markov chain Monte Carlo settings
☒	☐	For hierarchical and complex designs, identification of the appropriate level for tests and full reporting of outcomes
☐	☒	Estimates of effect sizes (e.g. Cohen's *d*, Pearson's *r*), indicating how they were calculated

Our web collection on statistics for biologists contains articles on many of the points above.

Software and code

Policy information about availability of computer code

Data collection	RNA sequencing was performed at Oxford Genomics. Peptides were separated by nanoflow ultra-high pressure liquid chromatography (UPLC, NanoAcquity, Waters) and analyzed by mass spectrometry using a LTQ-Orbitrap XL mass spectrometer (Thermo Fisher Scientific).
Data analysis	CIBERSORT, CIBERSORTX, xCELL, RStudio (2022.02.3+492 "Prairie Trillium"), R (version 4.1.1), and GraphPad Prism software version 8.3.0 analysis packages were used for analysis of datasets. Proteomic raw reads were aligned using MASCOT databases. Quantification of fiber diameter and alignment was performed on SEM micrographs using ImageJ version 1.50i (NIH).

For manuscripts utilizing custom algorithms or software that are central to the research but not yet described in published literature, software must be made available to editors and reviewers. We strongly encourage code deposition in a community repository (e.g. GitHub). See the Nature Portfolio guidelines for submitting code & software for further information.

Data

Policy information about availability of data

All manuscripts must include a data availability statement. This statement should provide the following information, where applicable:

- Accession codes, unique identifiers, or web links for publicly available datasets
- A description of any restrictions on data availability
- For clinical datasets or third party data, please ensure that the statement adheres to our policy

The RNAseq data have been deposited in NCBI's Gene Expression Omnibus under the GEO Series accession number GSE186145 (https://www.ncbi.nlm.nih.gov/geo/query/acc.cgi?acc= GSE186145).

The mass spectrometry proteomics data have been deposited to the ProteomeXchange Consortium via the PRIDE partner repository with the dataset identifier PXD036940 and 10.6019/PXD036940.

The raw HGSOC count per million (CPM) RNA-seq dataset (GSE71340) collected in a previous study, was downloaded from the NCBI Gene Expression Omnibus (GEO) database and protein mass ratios (PXD0040601) from the PRIDE database. Corresponding HGSOC cellularity data, obtained by manually counting six major leukocyte subtypes was obtained from the CanBuild site (canbuild.org.uk).

Human research participants

Policy information about studies involving human research participants and Sex and Gender in Research.

Reporting on sex and gender	only tissue samples from females were used in this work.
Population characteristics	Patient age, chemotherapy status, tumour stage and BRCA status is recorded within supplemental dataset 5.
Recruitment	n/a study based on existing samples.
Ethics oversight	Metastatic ovarian cancer patient samples were kindly donated by women undergoing surgery at Barts Health NHS trust between 2010 and 2017. All tissue obtained was deemed by a pathologist to be surplus to diagnostic and therapeutic requirements and was collected under the terms of Barts Tissue Bank (HTA license number 12199. REC no: 10/H0304/14). Each patient gave written informed consent. The work was conducted in accordance with the Declaration of Helsinki and International Ethical Guidelines for Biomedical Research Involving Human Subjects (CIOMS).

Note that full information on the approval of the study protocol must also be provided in the manuscript.

Field-specific reporting

Please select the one below that is the best fit for your research. If you are not sure, read the appropriate sections before making your selection.

☒ Life sciences ☐ Behavioural & social sciences ☐ Ecological, evolutionary & environmental sciences

For a reference copy of the document with all sections, see nature.com/documents/nr-reporting-summary-flat.pdf

Life sciences study design

All studies must disclose on these points even when the disclosure is negative.

Sample size	The sample size was based on power calculations from previous data collected (Pearce et al, Cancer Discovery 2018). We estimated a minimum threshold of 28 tissues.
Data exclusions	We have excluded sample data in two places within the main manuscript where the reason for exclusion is explained. Briefly, in one case this was due to the proteomics data for one sample that clustered far away from all other samples, and one sample was not used for immune infiltrate analysis because there was not enough material to conduct all research on this tissue.
Replication	In general each experiment had a minimum of two technique replicates (usually three) and three experimental replicates. For the proteomics analysis the tissue samples were run in duplicate and the value averaged.
Randomization	Tissue samples were grouped based on hierarchal clustering extracted from the proteomics dataset.
Blinding	No blinding of the data was used because this was an investigatory study.

Reporting for specific materials, systems and methods

We require information from authors about some types of materials, experimental systems and methods used in many studies. Here, indicate whether each material, system or method listed is relevant to your study. If you are not sure if a list item applies to your research, read the appropriate section before selecting a response.

Materials & experimental systems

n/a	Involved in the study
	☒ Antibodies
	☒ Eukaryotic cell lines
☒	Palaeontology and archaeology
☒	Animals and other organisms
☒	Clinical data
☒	Dual use research of concern

Methods

n/a	Involved in the study
☒	ChIP-seq
	☒ Flow cytometry
☒	MRI-based neuroimaging

Antibodies

Antibodies used

Antibodies. The following antibodies were used for immunohistochemical analyses: anti-PAX8 (clone BC12, ab124445; 1:1000; Abcam), anti-FOXP3 (clone eBio7979 (221D/D3), 14-7979-82; 1:1500; Invitrogen, UK); anti-CD4 (clone 4B12, M7310; 1:300; Dako), anti-CD8 (clone C8/144B, ab75129; 1:500; Abcam), anti-CD68 (clone PG-M1; GA613; 1:12000; Dako), anti-CD163 (clone 10D6, MA5-11458; 1:1000; Thermo Fisher), anti-CD20cy (clone L26; M0755; 1:400; Dako), anti-VCAN (polyclonal, Ab202906; 1:200; Abcam), anti-COL1A1 (polyclonal, HPA011795; 1:500; Sigma Aldrich), anti-FN1 (polyclonal, Ab23750; 1:500; Abcam), anti-CTSB (ab125067; 1:50; Abcam), anti-CS (clone CS-56, Ab11570; 1:600; Abcam).

The following antibodies were used for monocyte-derived macrophage flow cytometry analyses: CD206 (FITC, 1:100, Cat: 321104, Biolegend), HLA-DR (AF700, 1:100, Cat: 307626, Biolegend), CD45 (PerCP, 1:100, Cat: 368506, Biolegend), CD209 (APC, 1:100, Cat: 330107, Biolegend), CD47 (AF700, 1:100, Cat: 323125, Biolegend), CD36 (BV421, 1:100, Cat: 336229, Biolegend), CD38 (BV605, 1:100, Cat: 356641, Biolegend), CD86 (BV650, 1:100, Cat: 305428, Biolegend), CD163 (PE, 1:100, Cat: 333606, Biolegend), CD11b (AF594, 1:100, Cat: 301340, Biolegend), CD14 (PE-Cy5, 1:100, Cat: 15-0149-42, Invitrogen), and CD204 (PE-Cy7, 1:100, Cat: 371907, Biolegend).

K562 cells were centrifuged and resuspended in RPMI at 5 x 105 cells/mL and plated onto an ultra-low attachment plate at 25,000 cells/well and incubated with anti-CD47 antibody (Cat: 16-0479-85, ThermoFisher).

The following antibodies were used for T cell flow cytometry analyses: CD223-FITC (1:50, 3DS223H, ThermoFisher), CD8-APC (1:100, SK1, Biolegend), CD366-APCCy7 (1:50, F38-2E2, Biolegend), CD4-BV711 (1:100, SK3, BD Bioscience), CD3-PE (1:100, UCHT1, Biolegend) and CD279-PECy7 (1:20, EH12.1, BD Bioscience).

T cells were activated with 1 μg/ml anti-CD3 (Clone: OKT3, Cat: 317326, Biolegend) and 5 μg/ml anti-CD28 (Clone: CD28.2, Cat: 302943, Biolegend).

Validation

All antibodies were validated for their use in the application and species according to manufacturer's websites. IHC antibodies were validated as compared to an isotype control. Flow antibodies were validated by titration.

Eukaryotic cell lines

Policy information about cell lines and Sex and Gender in Research

Cell line source(s)	K-562 (ATCC CCL-243) cell line originally supplied by ATCC.
Authentication	STR profiling performed by ATCC.
Mycoplasma contamination	All cell lines tested negative for mycoplasma contamination.
Commonly misidentified lines (See ICLAC register)	None.

Flow Cytometry

Plots

Confirm that:

☒ The axis labels state the marker and fluorochrome used (e.g. CD4-FITC).

☒ The axis scales are clearly visible. Include numbers along axes only for bottom left plot of group (a 'group' is an analysis of identical markers).

☒ All plots are contour plots with outliers or pseudocolor plots.

☒ A numerical value for number of cells or percentage (with statistics) is provided.

Methodology

Sample preparation

For flow cytometry analysis, monocytes were isolated from anonymised blood samples and cultured on decellularised tissue to educate a macrophage phenotype. They were initially washed using PBS and then stained with 1:1000 Zombie NIR in PBS

	supplemented with Fc receptor and monocyte blocker (Biolegend) for 20 minutes at RT. Cells were then washed and stained in 50µL total volume PBS, 2% BSA, 2mM EDTA (FACS buffer) using an antibody master mix for 20 minutes at 4°C. Subsequent stained cells were washed in FACS buffer and analysed.
Instrument	Cytofluorimetric analysis was performed using a 4-laser Fortessa flow cytometer (BD).
Software	Data was analysed using Flowjo.
Cell population abundance	For macrophages 2,000 live events were collected and for T cells 5,000 live events were selected by Zombie NIR gating strategy. The abundance of relevant cell populations include: 84.9% Live Cells selected by Zombie NIR gating, 99.8% CD45+ selected using a CD45 FMO control, and 99.1% CD45+ CD14+ using a CD14 FMO control.
Gating strategy	Gating strategy: preliminary FSC/SSC gates were set at 10K and 30K, respectively. Subsequently, live cells were gated using Zombie NIR (Gate set at 10^4.5). Macrophages were gated as CD45+ (Gate set at 10^3) and CD14+ (Gate set at 10^3).

☒ Tick this box to confirm that a figure exemplifying the gating strategy is provided in the Supplementary Information.
