# Supplementary material for: Extracellular matrix educates an immunoregulatory tumor macrophage phenotype found in ovarian cancer metastasis
Source: Nat Commun. 2023 May 15;14:2514. doi: 10.1038/s41467-023-38093-5 (PMC10185550; doi:10.1038/s41467-023-38093-5)
Supplement: Supplementary file 4 — Description of Additional Supplementary Files [file 41467_2023_38093_MOESM4_ESM.docx]

**Description of Additional Supplementary Files**

Supplementary Data 1

Description: IHC immune cell markers used to verify deconvolution methods

Supplementary Data 2

Description: Spearman correlative analysis of CIBERSORTx, matrisome transcriptomics and matrisome proteomics for 32 HGSOC omental samples.

Supplementary Data 3

Description: Spearman correlative analysis of xCell, matrisome transcriptomics and matrisome proteomics for 32 HGSOC omental samples.

Supplementary Data 4

Description: Reference signatures used for M0, M1 and M2 macrophages from CIBERSORT, CIBERSORTx (Tab: CIBERSORT LM22) and xCell (Tab: xCell).

Supplementary Data 5

Description: Library of ovarian cancer tissue samples.

Supplementary Data 6

Description: Proteomics analysis of 39 ovarian cancer omental samples.

Supplementary Data 7

Description: Disease score analysis of 39 ovarian cancer samples.

Supplementary Data 8

Description: IHC immune cell counts.

Supplementary Data 9

Description: Gene expression in log2(TPM+1) related to Figure 4B and 4H.

Supplementary Data 10

Description: LENGENDPLEX analysis of decellularized tissue cultured macrophage chemokines.

Supplementary Data 11

Description: List of genes per WGCNA cluster related to Figure 5A.

Supplementary Movie 1

Description: Representative video of laser capture dissection. The video shows tumor areas being cut and selected on a PALM dissection microscope.
